# Single-cell atlases of two lophotrochozoan larvae highlight their complex evolutionary histories

**DOI:** 10.1126/sciadv.adg6034

**Published:** 2023-08-02

**Authors:** Laura Piovani, Daniel J. Leite, Luis Alfonso Yañez Guerra, Fraser Simpson, Jacob M. Musser, Irepan Salvador-Martínez, Ferdinand Marlétaz, Gáspár Jékely, Maximilian J. Telford

**Affiliations:** ^1^ Centre for Life’s Origins and Evolution, Department of Genetics, Evolution and Environment, University College London, London WC1E 6BT, UK.; ^2^ Living Systems Institute, University of Exeter, Stocker Road, Exeter, UK.; ^3^ Developmental Biology Unit, European Molecular Biology Laboratory, 69117 Heidelberg, Germany.

## Abstract

Pelagic larval stages are widespread across animals, yet it is unclear whether larvae were present in the last common ancestor of animals or whether they evolved multiple times due to common selective pressures. Many marine larvae are at least superficially similar; they are small, swim through the beating of bands of cilia, and sense the environment with an apical organ. To understand these similarities, we have generated single-cell atlases for marine larvae from two animal phyla and have compared their cell types. We found clear similarities among ciliary band cells and between neurons of the apical organ in the two larvae pointing to possible homology of these structures, suggesting a single origin of larvae within Spiralia. We also find several clade-specific innovations in each larva, including distinct myocytes and shell gland cells in the oyster larva. Oyster shell gland cells express many recently evolved genes that have made previous gene age estimates for the origin of trochophore larvae too young.

## Teaser

Single-cell atlases of platyhelminth and mollusc larvae reveal homologous cell types and phylum-specific innovations.

## INTRODUCTION

**Fig. 1.**
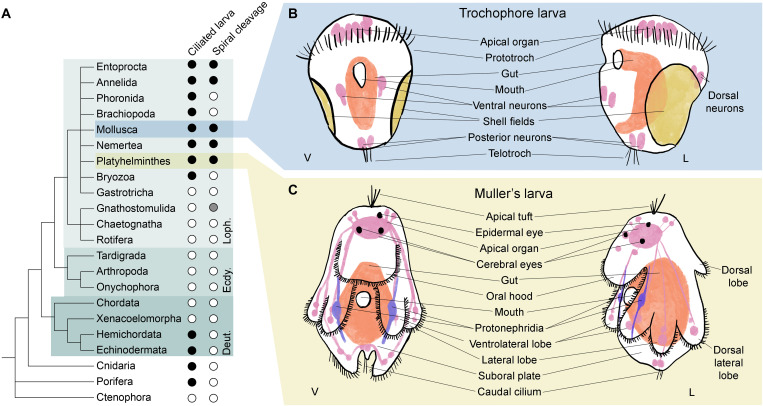
Larvae are a common feature of Metazoa. (**A**) A phylogeny of animals shows the widespread presence of ciliated larvae, especially among the superphylum Lophotrochozoa (Loph.). Black dots, character presence; white dots, absence; gray dots, unconfirmed. (**B** and **C**) Schematics of larvae presented in this study, (B) in blue box the trochophore larva of the Pacific oyster *Crassostrea gigas*, represented as both ventral (V) and lateral (L) views. *C. gigas* larvae at the trochophore stage lack an apical tuft of sensory cilia and paired protonephridia. (C) In yellow, schematics of the Müller’s larva of the polyclad flatworm *Prostheceraeus crozieri* depicted as ventral and lateral views.

Indirect development via a small, pelagic, and ciliated larva is found in some members of at least 12 metazoan phyla (Fig. 1A). Ciliated larvae are generally understood to enhance dispersal in aquatic animals. The larval stage, which may last several weeks, is typically followed by a more or less rapid metamorphosis into a very different adult form. Six of the metazoan phyla with ciliated larvae—molluscs, annelids, nemerteans, brachiopods, phoronids, and flatworms—are grouped within the super phylum of the Lophotrochozoa. Spiral cleavage is a second important shared characteristic among most of these phyla, supporting the monophyly of the Spiralia within the Lophotrochozoa. The most iconic of these larvae, to which the others are often compared, is the trochophore larva found in the annelids and molluscs.

Trochophore larvae get their name from their preoral ciliary band or prototroch that is situated as an equatorial girdle dividing the animal approximately in to two. In a canonical trochophore larva (e.g., an annelid), eyes are found on the anterior episphere and, at the anterior pole of both typical molluscan and annelid trochophores, there is a long tuft of sensory cilia called the apical tuft (*1*). Posterior to the prototroch is the mouth and additional ciliary bands may be found more posteriorly including a terminal telotroch (*2*). Internally, a canonical trochophore larva has an apical organ below the apical tuft, paired protonephridia, and a larval gut (see Fig. 1B).

While lophotrochozoan larvae from phyla outside the molluscs and annelids have been considered to be derived forms of the canonical trochophore, these larvae have also been suggested to be cases of convergent evolution driven by similar selective pressures (*3*, *4*). The Müller’s larva of the polyclad flatworms shares with trochophores a locomotory ciliated band, anterior eyes, an apical tuft of cilia, an apical organ, and protonephridia but, in other ways, differs from the trochophore (*4*). In particular, the body of the Müller’s larva is more complex; its ciliary band runs a convoluted path along the edges of eight lobes of the body (two ventrolateral, two lateral, two laterodorsal, one oral, and one dorsal lobe) and is used both for filter feeding and for locomotion (see Fig. 1C).

The existence of both shared larval characters and clade-specific differences has prompted heated debates regarding lophotrochozoan larval evolution such as how often larvae have evolved, what the adaptive advantages of larvae in different groups are, and what the developmental and genetic underpinnings of the evolution of different larval types are (*5*). Comparative efforts to understand the evolution of lophotrochozoan larvae have so far focussed on morphological, developmental, and (sparse) molecular comparisons of putatively homologous shared structures (such as ciliary bands or apical organs) (*6*–*10*). For nearly all of these larvae, however, we still lack a thorough molecular characterization of larval anatomy, and this would represent an essential contribution to this debate. We do not yet know, for example, how many cell types larvae have, whether any of these cell types are common between larval types, and the extent to which different larvae may have unique cell types or larval organs.

Historically, cell morphology and spatial position within a tissue were used to characterize cell types (*11*). Single-cell sequencing now allows us to obtain the full transcriptional signature of single cells and to group these together by similarity in cell clusters. Cell clusters can be used as a proxy for cell types. Some authors have proposed that cell types should be individuated by distinct transcription factors (TFs) modules and that they can be hierarchically organized in cell clades or families (*11*, *12*). The expression levels of genes specific to certain cell types or cell families can then be compared across species to ask whether they have cell types in common (*13*–*16*).

Here, using single-cell sequencing, we have characterized the cellular component of two distinct lophotrochozoan larvae: the trochophore larva of the Pacific oyster *Crassostrea gigas* and the Müller’s larva of the tiger flatworm *Prostheceraeus crozieri*. We have described multiple cell types in each larva and located these spatially using in situ hybridization (ISH) and hybridization chain reaction (HCR), thereby revealing the complex anatomy of both larvae (*17*). By considering the phylogenetic ages of expressed genes in different cell types, we found that larvae appear to be made up of a combination of ancient and more recently evolved cell types that coexist in the larval body. Last, we attempted to identify cell types that might be homologous between the oyster trochophore and flatworm Müller’s larvae and have extended this comparison to the cell types of the primary larva of a sea urchin (*18*). These comparisons highlighted molecular similarities between ciliary bands and apical organs of trochophores and Müller’s larva, which might indicate that these larvae are homologous.

## RESULTS AND DISCUSSION

### A single-cell atlas for Pacific oyster trochophore larva

**Fig. 2.**
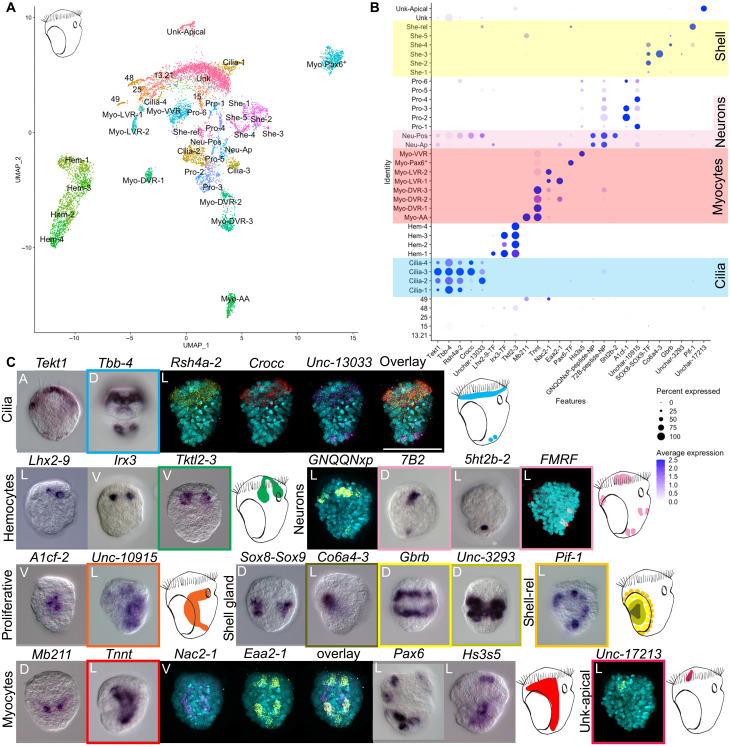
Cell atlas of the trochophore larva of *C. gigas*. (**A**) Two-dimensional uniform manifold approximation and projection (UMAP) showing cell clusters for the oyster larva. (**B**) Dotplot of marker gene expression for different cell clusters: yellow highlights the shell gland clusters, pink highlights the neuronal clusters, red highlights the myocyte clusters, and blue highlights the ciliary clusters. Dotplots show expression of genes (*x* axis) in each cell cluster (*y* axis) of the oyster scRNA-seq. Shades of blue indicate average expression, and the size of dots indicates the percentage of cells expressing the gene. (**C**) ISH of the marker genes shown in (B) with schematic of expression in the larva (color of the square indicates which gene/s was/were used for each schematic). A, apical view; P, posterior view; V, ventral view; D, dorsal view; L, lateral view with mouth on the right. HCR expression without 4′,6-diamidino-2-phenylindole in a larger format is available in fig S1. Scale bar, 50 μm.

We generated a cell atlas of the trochophore larva of the Pacific oyster—a commercially important species readily available from fishmongers. This species has a chromosome-scale genome assembly available and is becoming a popular model to study larval evolution (*19*–*21*). After in vitro fertilization to produce trochophore larvae, we carried out four rounds of cell capture from three distinct dissociation experiments using the 10x Chromium single-cell RNA sequencing (scRNA-seq) system. Initial shallow sequencing allowed us to select the best-quality library (see fig. S2), which was then sequenced further to obtain a final dataset of 8597 cells expressing a total of 26,275 genes with a mean number of unique molecular identifiers (UMIs) of 30,000 per cell. Oyster larvae contain fewer than 500 cells, and our data therefore represent an approximately 17-fold coverage of each larval cell, suggesting that even rare cell types have been sampled. By applying dimensionality reduction, we identified a total of 37 cell clusters organized into six cell families: ciliary cells, shell field cells, neurons, myocytes, hemocytes, and proliferative cells (fig. S3). These include four ciliary cell clusters, two neuronal cell clusters, eight myocyte clusters, six shell related clusters, four hemocyte clusters, and six proliferative cell clusters (see Fig. 2A). To validate these and to assign putative identities to cell families and specific clusters of interest, we selected differentially expressed gene markers and used a combination of literature searches, chromogenic ISH, and in situ HCR (*17*) (see Fig. 2, B and C).

#### Oyster trochophore ciliary bands and a reduced apical organ

Notable features of most marine invertebrate larvae are their ciliated bands, which have been proposed to be homologous across larval types (*6*, *10*, *22*, *23*). From a morphological point of view, at the stage analyzed here, the trochophore larva of the Pacific oyster has a prototroch and a small telotroch. We recovered the expression of previously published ciliary markers *tubulin-beta-chain* (*tbb-4*) and *tektin* (*tekt1*) (*24*) in four clusters. ISH for these genes shows expression in both the prototroch and the telotroch (Fig. 2, B and C). HCR of the marker gene for cluster Cilia-3 and Cilia-4 rootletin (crocc) shows staining mostly in the prototroch (see Fig. 2, B and C, and fig. S4). In contrast, the marker gene for cluster Cilia-2 Unchar-13033 is expressed strongly in the telotroch and in some cells posterior to the prototroch (Fig. 2C and fig. S4). The four ciliary clusters share 59 markers, and some may be separate cell subtypes (see figs. S1 and S4). Cluster Cilia-2 is characterized by the expression of several specific TFs including *ascl4*, *pax2/5*, *dbx*, *gsc2*, and *foxL1*, while the TFs *plscr1-1* and *mafb* are specific to clusters Cilia-3 and Cilia-4 (fig. S4). At the stage analyzed here, the larva does not have an apical ciliary tuft.

A second feature typical of trochophore larvae is their apical organ. Previous experiments using immunocytochemistry against FMRFamide, serotonin, and vesicular acetylcholine transporter (VAChT) in the oyster larva have identified a small apical organ made up of five or six apical neurons. The rest of the nervous system is composed of a pair of dorsal neurons, a pair of ventral neurons, and a pair of posterior neurons (*25*). To identify neurons in our scRNA data, we searched for cells expressing neuropeptide (NP) precursors and a suite of additional neuronal markers (Fig. 2, B and C, and fig. S5; a full list of characterized NPs is available in table S1). We found two clusters of potential neurons that express the neuronal markers: voltage-gated sodium channel (*scna1*), voltage-dependent calcium channel subunit alpha-1 (*cac1a-1*), two synaptotagmins (*sy65-1* and *sy65-2*) and the proneuropeptides *GNQQNxP*, *allatotropin*, and *7B2* (see Fig. 2C and fig. S5). ISH and HCR for marker genes from these two neuronal clusters revealed that one cluster corresponds to apical and dorsal neurons (Neu-Ap) and the second corresponds to posterior neurons (Neu-Pos) (see Fig. 2, B and C, and fig. S1). We identified two ventral neurons by HCR against the *FMRFamide* precursor mRNA; however, these ventral neurons are represented by only eight cells in our scRNA-seq data and these do not form a separate cluster (Fig. 2, *FMRF* staining, and fig. S1). Proneuropeptides restricted to the apical neurons are *myomodulin* (which is restricted to only one or two cells of the apical organ) (fig. S1), *CCWamide*, *corazonin*, and *allatostatin* (fig. S5). The TFs *prd6*, *awh2*, *hbn*, *tbx5*, *prox*, *delta*, *sox2*, and *zic1/2/5/4* are specific to apical neurons (fig. S5). *Awh*, *hbn*, *delta*, and *zic* are also found in the apical organ of the sea urchin larva and in the nervous systems of several other bilaterians (*26*). Posterior neurons are characterized by the expression of *TRH*, *MIP*, *AKH-GNRH*, *pedal peptide 1*, *CCAP-peptide*, and *whitnin-PKYMDT-proctolin* precursors (fig. S5). The expression of the TFs *barH2*, *prop*, *pou4*, and *evx* is also specific to the posterior cluster.

Overall, the oyster trochophore larva has an extremely reduced apical organ, made of only five to six cells with similar transcriptional signatures (*27*). A few other neurons (two dorsal, two ventral, and two posterior) are found. It is possible that the low degree of neuronal complexity is due to the larva’s small body size (~50 μm) and the early stage considered here. None of the clusters analyzed showed protonephridial staining, and classical protonephridial marker genes (e.g., *pou3*, *sall*, and *lhx1/5*) are not expressed in any cluster (*28*). Protonephridia were also not found in previous studies using immunohistochemistry against acetylated tubulin (*25*). It is possible that protonephridia appear at a later developmental stage.

#### Multiple distinct muscle types in oyster larvae

A search for muscle and myocyte-related genes revealed a previously undescribed complexity in the muscular system of the oyster larva. A total of 1935 cells expressed multiple myocyte and muscle markers [including *tropomyosin*, *troponin-t* (*tnnt*), *twitchin*, and several *myosin chain* proteins] as well as various TFs (including several GATA and Fox family genes) (Fig. 2 and figs. S3 and S6). The myocytes could be separated into eight clusters (fig. S3), and these could be associated with five morphologically distinct muscles within the larva based on ISH and HCR stainings: anterior adductor muscles (Myo-AA), dorsal velum retractor muscles (Myo-DVR), ventral velum retractor muscle clusters (Myo-VVR), larval retractor muscles (Myo-LVR), and an enigmatic group of myocytes expressing *pax6* (Myo-Pax6^+^).

*Protein mab-21-like* (*mb211*), a marker of the Myo-AA cluster, is expressed on either side of the hinge of the forming shells, consistent with the location of the anterior adductor muscles; these are a bivalve innovation that control the opening and closing of the shell plates (Fig. 2, B and C) (*29*). Cells of the Myo-AA cluster coexpress both *paramyosin* and *calponin*, a combination that is typical of mollusc catch muscles which can maintain passive tension for long periods of time with minimal energy requirements. Catch muscles are found in other invertebrates such as insects, crayfish, nematodes, and brachiopods; in bivalves, this muscle is used to keep their shell closed (*30*).

ISH for the marker gene *tnnt* revealed expression in two roughly symmetrical patches on the anterior part of the trochophore corresponding to the location of the dorsal velum retractor muscles (DVR) (*31*). The cluster-specific marker *heparan sulfate glucosamine 3-O-sulfotransferase 5* (*hs3s5*) showed expression on the ventral side in the region of the ventral velum retractor muscles clusters (VVR). The two markers, *excitatory amino acid transporter 2* (*eaa2-1*) and *sodium/calcium exchanger 2* (*nac2-1*), highlighted a cross-like pattern on the dorsal part of the larva consistent with the position of the larval retractor muscles (LVR) (Fig. 2, B and C) (*32*).

Last, we identified a cluster (Myo-Pax6^+^) expressing several myocyte markers such as *troponin C* (*tnnc*), *tubulin-beta-chain* (*tbb-1*), *actin* (*act-1*), *myosin light chain kinase* (*mylk-2*), and *titin* (see fig. S7). Cells of this cluster also expressed several so-called “eye master regulators” such as *pax6*, *eya*, and *six1/2*, although no opsin expression was identified in this cluster (fig. S7). ISH of the cluster-specific marker *pax6* showed expression in cells scattered symmetrically across the larva (Fig. 2, B and C). This is not the first instance where *pax6*, *eya*, and *six1/2*, as well as other so-called eye master regulators, are found in non–photoreceptor-type cells—these same TFs are found in the larval hydropore canal and coelomic pouches of sea urchins, as part of kidney development and the specification of somitic muscle in vertebrates, as well as in several tissues in amphioxus (*33*).

#### The innovation of shell gland cell types

A key character of the phylum Mollusca is the shell. A search for previously described shell gland markers such as *tyrosinase* (*tyro*), *mantle protein* (*mp*), and *nacrein* (*manl-9*) highlighted six clusters (*34*–*36*). Specific markers for these clusters show concentric rings of expression in or around the two dorsally located, paired shell glands (see Figs. 1B and 2C). Specifically, a marker for the clusters She-1 and She-2 (*Unchar-1938*) stains the inner area of the shell gland; the marker for cluster She-3, *collagen alpha-6*(*IV*) *chain* (*co6a4-3*), stains the hinge between the two presumptive shells; and a marker for She-4 and She-5 [*gamma-aminobutyric acid receptor subunit beta* (*gbrb*)] stains the outer layer of the shell gland. Lastly, the marker of the cluster She-rel, *ATP-dependent DNA helicase PIF1* (*pif-1*), stains the outermost layer. It is possible that some of the inner cells are responsible for secreting the prodissoconch I (the two D-shaped larval shells) and that the more external cells will secrete the prodissoconch II (the veliger shell) at a later stage (*37*).

**Fig. 3.**
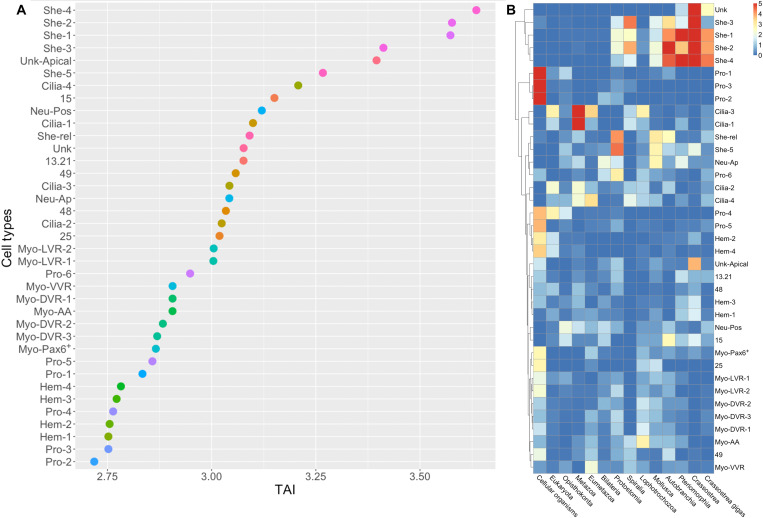
Gene age analyses in different cell types of the oyster larva show that shell gland cells have a young gene signature. (**A**) Transcriptome age indices (TAI) for different cell types; smaller TAI values correspond to “older” gene ages. Gene age is inferred using a phylostratigraphy approach; the TAI is then calculated on the log-transformed gene average expression per cluster. (**B**) Heatmap showing enrichment test −log_10_(*P* value) for marker genes phylostrata per cell type in the oyster. Enrichment was computed using a hypergeometric test applied to the number of marker genes in each cluster per phylostrata compared to the global set of expressed genes.

Stage-specific bulk RNA-seq has recently been used to explore the expression of phylogenetically older and younger genes throughout the development of lophotrochozoan larvae. This analysis revealed a peak of expression of young genes in the trochophore stage of the Pacific oyster and the scallop *Pecten yessoensis* (*5*, *20*, *21*). These findings were interpreted as indicating that the mollusc trochophore was an evolutionarily recent innovation and arguing against the idea that the trochophore larva is the phylotypic stage of molluscs (*20*). We extended this phylostratigraphic approach into a cell-type level of resolution by computing the phylogenetic ages of genes expressed in each characterized cell type of the oyster larva. We found that the expression of very young genes was restricted to the shell gland cell types, similarly to what was found in adults (*38*). This causes the shell gland to have the highest (youngest) transcriptome age index (TAI; see Fig. 3, A and B). Our result indicates that the peak in TAI observed in previous studies is likely an artefact caused by the innovation of a shell added to an older larval body. Alternatively, the shell producing cells may have undergone more rapid evolution than the rest of the larval body, producing a subset of cells with a TAI that is younger than the rest of the larva. Our result highlights the benefit of looking at gene expression at the cell-type level rather than in bulk. The lowest (oldest) TAI is found in cell types such as proliferative cells and hemocytes (Fig. 3A). For a description of hemocytes and proliferative cell clusters as well as TAI results in the flatworm larva, see figs. S3 and S8.

### A single-cell atlas of the polyclad flatworm Müller’s larva

**Fig. 4.**
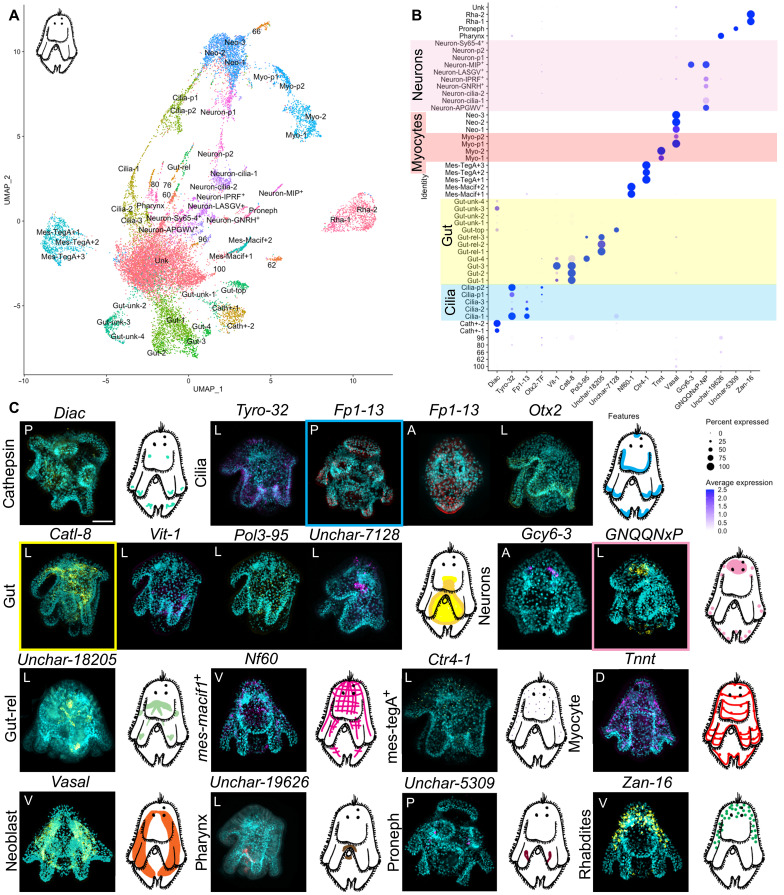
Single-cell atlas of the Müller’s larva of *P. crozieri*. (**A**) UMAP showing cell clusters for the polyclad flatworm larva. (**B**) Dotplot of marker gene expression for cell clusters: yellow highlights the gut clusters, pink highlights the neuronal clusters, red highlights the myocyte clusters, and blue highlights the ciliary clusters. Dotplot graphs show expression of genes (*x* axis) in each cell cluster (*y* axis) of the flatworm scRNA-seq: Shades of blue indicate average expression, and the size of the dots indicates the percentage of cells expressing the gene. (**C**) HCR of the marker genes shown in (B) with schematics of their expression in the larva (color of the square indicates which gene/s was/were used for schematic). A, apical view; P, posterior view; V, ventral view; D, dorsal view; L, lateral view with mouth on the left. Images of HCR expression without 4′,6-diamidino-2-phenylindole and in larger format is available in fig S9. Scale bar is 50 μm.

To generate the Müller’s larval cell atlas, we carried out four rounds of cell capture from two separate dissociation experiments using 10x Chromium scRNA-seq. In-depth sequencing of the best libraries produced a final dataset of 17,605 cells expressing a total of 33,305 genes with over 40,000 mean UMIs per cell (see fig. S10). Müller’s larvae are three times larger than oyster larvae and are made up of ~2000 cells, so, in our datasets, each cell should be represented ~8 times. Dimensionality reduction allowed us to identify a total of 51 distinct cell clusters that can be grouped into seven cell families: myocytes, digestive cells, ciliated cells, neurons, secretory cells, proliferative cells, and a group of flatworm-specific cells (fig. S11 and Fig. 4A). To validate cluster identities, we used literature searches, in situ HCR, and comparisons with the published cell atlas of the adult planarian worm *Schmidtea mediterranea* (*32*).

#### The simple muscles and complex gut of the Müller's larva

A search for myocyte markers in our scRNA-seq data, such as *troponin-i* (*tnni*), *troponin-t* (*tnnt*), *tropomyosin* (*tpm2*), *titin*, and *paramyosin*, revealed expression in four clusters. HCR of the general marker *tnnt* stained the larval muscles and resembles what has previously been shown using immunohistochemistry (*2*) (Fig. 4C). Of these four clusters, two probably represent myocyte precursors, as they share markers with neoblast clusters, while the other two (Myo-1 and Myo-2) share over 70 marker genes, suggesting that they could represent myocyte subtypes and/or differentiating states. This indicates that the Müller’s larva has a much simpler muscle cell-type complement than the mollusc trochophore.

Contrary to what we found in the oyster, at least eight cluster markers showed expression in or around the gut. These include a cluster of cells near the top of the gut (*Unchar-7128*), a cluster of cells localized in the posterior part of the gut [*vitellogenin-1* (*vit-1*)] and scattered cells in the gut lining [*DNA polymerase delta catalytic subunit 3* (*pol3-95*)] (Fig. 4, B and C, and fig. S9). We also found three clusters (Gut-rel-1, Gut-rel-2, and Gut-rel-3) containing cells located in an inverted “V” around the pharynx, above the dorsal part of the gut and in the ventrolateral lobes (see expression of *Unchar-18205* in Fig. 4). These Gut-rel clusters group separately from the rest of digestive cells and will be discussed later (fig. S11). Lastly, we identified a cluster of pharyngeal cells highlighted by the flatworm-specific marker gene *Unchar-19626*. Pharyngeal cells are ciliated (*39*), and they group with ciliary bands cells and protonephridia (fig. S11). Unlike the oyster trochophore larva, the Müller’s larva has a clearly differentiated gut made up of several cell types with specific expression signatures and presumably distinct functions, similar to what has been shown in the planktonic larva of the sea urchin (*18*).

#### Trochophore-like features in the Müller’s larva

The possible homology of the ciliary bands of flatworm larvae and those of trochophores has long been debated as ciliary band ontogeny shows both similarities and differences (*3*, *4*). We found five clusters of ciliary band cells in the Müllers larva: two of these represent differentiating ciliary precursors and the other three differentiated ciliary clusters (fig. S11). The three differentiated ciliary clusters share only 16 marker genes. Cluster Cilia-1 is characterized by the expression of hundreds of specific markers (for a full list, see the Supplementary Materials), Cilia-2 is characterized by two specific caveolins (*cav1-8* and *cav1-9*) and the gene *enteropeptidase* (*entk*), while Cilia-3 shares most markers with the cluster Cilia-1. HCRs against the general markers tyrosinase (*tyro-32*), *otx-2*, and *adhesive plaque matrix protein* (*fp1-13*) show expression in the ciliated cells of the larval lobes as well as in the apical tuft (see Fig. 4 and fig. S9).

Another ciliated feature typical of trochophore larvae, which we did not find in the oyster, is their excretory organs: the protonephridia (fig. S11). Protonephridial markers such as *pou2/3*, *hunchback*, and *six1/2-2* allowed us to identify a cluster of protonephridial cells in the Müller’s larva (Fig. 4) (*28*). Cells from this cluster also express *cubulin* (specifically expressed in tubule cells of planarian protonephridia), *nephrin* (*nphn-1*), and *zonula occludens-2* (*zo2*) (*40*).

**Fig. 5.**
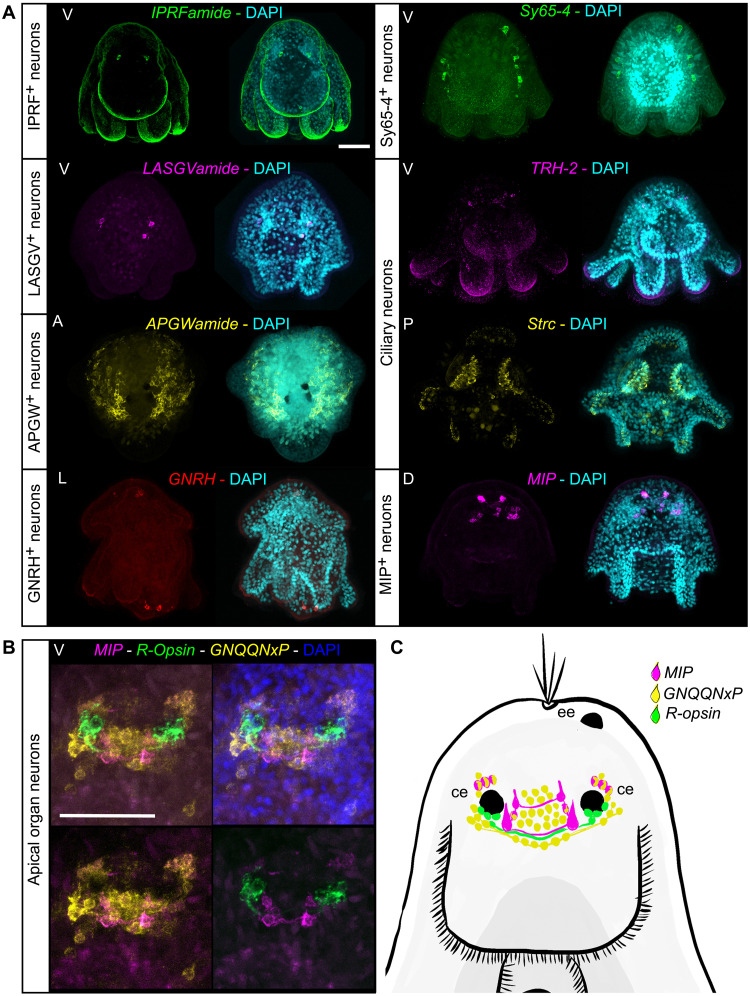
The complexity of the nervous system of the polyclad flatworm larva. (**A**) HCR staining of cluster markers and neuropeptides (NPs). (**B**) HCRs with a focus on apical organ neurons and (**C**) schematic drawing of the Müller’s larva apical organ. All HCR stainings are maximum projections. DAPI, 4′,6-diamidino-2-phenylindole; A, apical view; P, posterior view; V, ventral view; D, dorsal view; L, lateral view with mouth on the left; ee, epidermal eye; ce, cerebral eye. Scale bars, 50 μm.

Apical organs have also been proposed to be homologous between ciliated larvae (*7*, *41*) and we were therefore interested in identifying them in our datasets and comparing their genetic signatures. In the Müller’s larva, HCR against a shared marker for neuronal clusters (*GNQQNxP*) showed a large apical organ that sits around the ciliary eyes and several scattered neurons elsewhere in the larval body (see Figs. 4C and 5 and fig. S9). A combination of NPs and other neuronal markers allowed us to distinguish the presence of eight distinct neuronal clusters, each with its own signature combination of specific NPs (fig. S12; for a full list of NPs, see table S1).

We found a small cluster of neurons characterized by the expression of *IPRFamide* and *corazonin precursor genes*. *IPRFamide* precursor is expressed in two cells located apically and in one cell located in the suboral plate (Fig. 5, *IPRFamide* staining). A second cluster of anterior cells expresses *FMRFamide* and *LASGVamide precursor* genes as well as the TFs *Tbr1* (Fig. 5, *LASGVamide* staining). A third cluster contains *APGWamide*-positive cells (Fig. 5, *APGWamide* staining) that are localized in large symmetrical patches laterally on the top of the larva. Cells from this cluster are characterized by the expression of several other proneuropeptides: *APGWamide*, *GNQQNxP*, *calcitonin*, *allatostatin-A/buccalin*, *orexin/allatotropin*, and *7B2* (fig. S12). A fourth neuronal cluster contains *GNRH^+^* cells that show expression in a small patch near the apical tuft and in a couple of posterior neurons. Cells from this cluster also express the proneuropeptides *FVRIamide*, *FMRFamide*, *7B2*, *XHFamide*, *NKY1*, and *bursicon* (fig. S12).

We identified a fifth cluster of neurons that express several neuronal genes [including *sy65-4*, *cac1a*, *scna*, and a vesicular glutamate transporter (*vglu2*)] but no proneuropeptides (fig. S12). Cells from the sy65-4^+^ cluster also express the TFs *ETV4*, *repo*, and *prox-1*. The HCR staining for the marker gene *sy65-4* highlighted several cells bulging into the epidermal layer on each side of the apical part of the larva (Fig. 5).

In the apical organ, we found a cluster of neurons expressing a cocktail of proneuropeptides (*MIP*, *GNQQNxP*, *LSDWNamide*, and *7B2*); several neuronal markers such as *synaptotagmin*; the acetylcholine synthesizing enzyme *ChAT* and v*glu2*; and the TFs *sox14*, *HLF-1*, and *HLF-2* (fig. S12)*. HLF* is found in the apical organ of the sea urchin larva and in the nervous systems of several other animals (*26*). The cluster-specific marker *MIP* showed expression in two groups of cells posterior to the cerebral eyes and in a cluster of cells anterior to these. These four anterior cells, connected in pairs by neuronal processes (see Fig. 5) are reminiscent of the expression pattern of *MIP* in the annelids *Platynereis* and *Capitella* (*42*).

Two further clusters are of particular interest with regard to the potential homology of larval structures. These are identified as ciliary neurons and express several neuronal markers including *cac1a3*, *chat1*, *sy65-2*, and *vacht-1* as well as the proneuropeptides *LSDWNamide* and *TRH2* (fig. S12). These are likely to be the cilio-motor neurons described by Lacalli in another polyclad larva (*Pseudoceros canadensis*) and which are also found in *Platynereis* larvae, where they are thought to be a larval-specific character (*43*–*45*). Markers for these show expression in multiple cells in the ciliated lobes of the Müller’s larva (see Fig. 5), very similar to what has been seen in *Platynereis*. These markers are also expressed in a pair of apical cells. Equivalent cells are not found in the oyster trochophore.

Our results show that the Müller’s larva of a polyclad, described by some as a derived larva, presents many trochophore-like characters such as ciliary bands, protonephridia, cilio-motor neurons, and a complex apical organ. In the apical organ, we find a specific subset of *MIP^+^* cells similar to those found in other lophotrochozoan larvae (*42*).

#### Flatworm-specific cell types in the Müller’s larva

We found several clusters of potentially flatworm-specific cells. These include two clusters of rhabdites [secretory gland cells identified with the marker zonadhesin (*zan-16*)], cathepsin cells [identified with the marker *di-N-acetylchitobiase* (*diac*)], a cluster of cells that create a net-like structure in the mesenchyme [identified by the marker *neurofilament protein NF60* (*Nf60*)], and another cluster of scattered mesenchymal cells [*copper transport protein ctr4* (*ctr4-1*)]. Some of these mesenchymal cells may act together as part of an adhesive organ (involving secretion) like the one found in the flatworm *Macrostomum lignano* with which they share the expression of the marker intermediate filament gene *macif1* (*41*). We also found a large cluster of cells that express neoblast markers such as *vasa* and *piwi*. ISH for the gene *vasa* shows expression in the lateral mesenchyme of the animal. Their neoblast identity was also supported by comparing their molecular signature to that of the planarian flatworm *S. mediterranea* (for details, see fig. S13).

### Homologous cell types of oyster and flatworm larvae

**Fig. 6.**
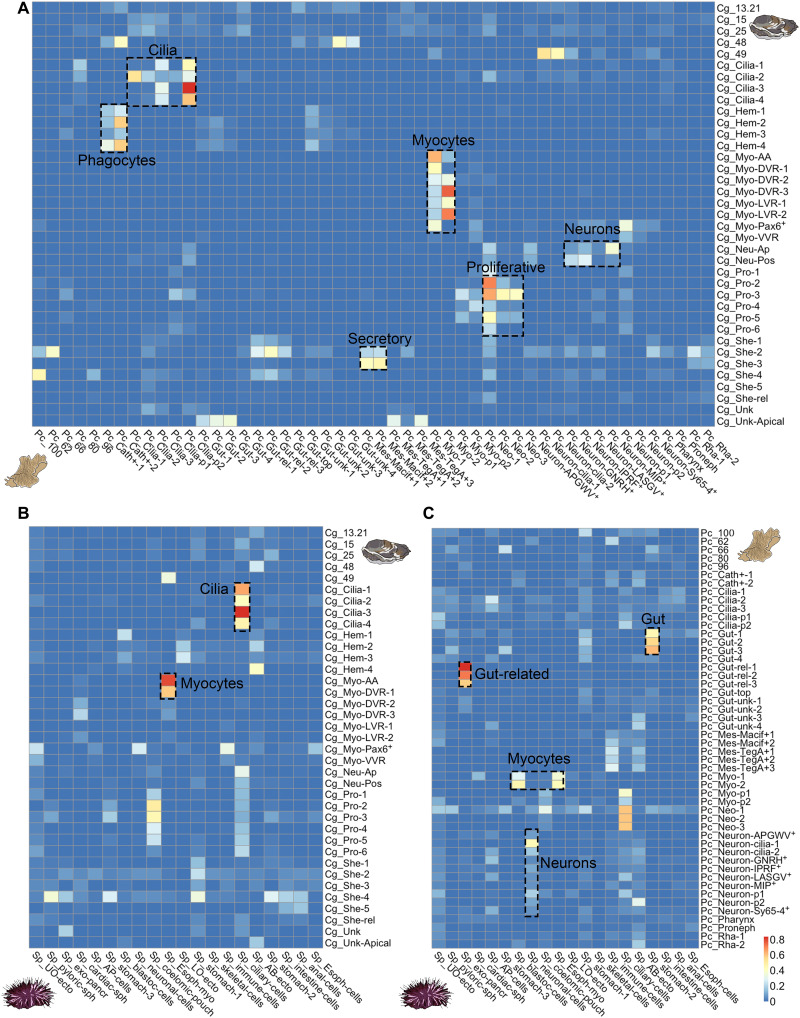
SAMap cell clusters alignment scores between invertebrate larvae. (**A**) Mapping of cell clusters between the trochophore larva of the Pacific oyster (Cg) and the Müller larva of polyclad flatworm (Pc). (**B**) Mapping of cell clusters between the trochophore larva of the Pacific oyster (Cg) and the pluteus larva of the sea urchin *Strongylocentrotus purpuratus* (Sp). (**C**) Mapping of cell types between the Müller larva of polyclad flatworm (Pc) and the pluteus larva of sea urchin (Sp). Alignment scores are defined as the average number of mutual nearest cross-species neighbors of each cell relative to the maximum possible number of neighbors (*13*).

We have shown that the trochophore larva of the Pacific oyster and the Müller’s larva of polyclad flatworms share several cell families such as myocytes, proliferative cells, ciliated cells, and neurons. To assess the potential homology of these cell families and/or cell types in the two larvae, we used SAMap to align and directly compare their molecular signature. SAMap uses the expression of homologous genes to align single-cell datasets across species (*13*) and identify shared markers. We found that, at the cell family level (figs. S3 and S11), myocytes, proliferative cells, ciliary band cells, and neurons aligned across species (see Fig. 6A). We also found unexpected matches between the hemocytes of the oyster and the cathepsin cells of the flatworm and between the shell gland cells of the oyster and the Macif1^+^ mesenchymal cells of the flatworm. In planarian flatworms, *cathepsin^+^* cells include glial cells, pigmented cells, and some cells with roles in phagocytosis (*46*); it is possible that flatworm cells with this latter function map with the oyster hemocytes. The alignment of oyster shell gland cells and flatworm-mesenchymal cells may reflect a common secretory role.

Notably, ciliary bands and apical organs are both larval-specific characters, while myocytes, proliferative cells, and hemocytes are potentially homologous across all Bilateria. For this reason, we further explored the matches between the ciliary cells and between neurons by looking at their shared molecular markers. SAMap found 133 oyster and 112 flatworm genes that are coexpressed between ciliary clusters (the difference in numbers is due to SAMap allowing many-to-many orthologs/paralogs matches). Many of these genes code for proteins known to be involved in the ciliary apparatus such as *annexins*, *calmodulins*, *rootletins*, *centrins*, and *calcineurins*; cilia and flagella associated proteins, *cubulins*, various *dyneins*, *enkurin*, *stomatin*, and *tektins*; as well as several genes that remain uncharacterized (for a full list, see fig. S14). Most of the effector genes do not seem to be shared across other ciliated tissues in the flatworm larva such as protonephridia or the cells of the pharynx so they are specific to ciliary bands (fig. S14). SAMap also recovered two shared TF markers (CEBPB 2 and 3 and CEBP-G), although these are not restricted to ciliary clusters (fig. S14).

TFs are often expressed at lower levels than effector genes, rendering them more difficult to detect as specific markers. We therefore performed a manual survey of all TFs (see fig. S15). We found several that were not detected as shared markers by SAMap (*dbx*, *gsc*, *otx*, *pbx*, *zfhx*, and *pax6*; fig. S15), which nevertheless have expression in ciliary band cells in both flatworm and oyster larvae. *Otx* is also expressed in the ciliary bands of larvae of the mollusc *Patella vulgata* (*46*), the annelid worm *Platynereis dumerilii* (*10*), the hemichordate *Ptychodera flava* (*47*), and in echinoderm larvae (*48*). It is worth noting that ciliary cells are the only cell types in the two larvae where we recovered shared homologous TF expression.

We also performed a manual investigation of spiralian-specific genes identified in a recent publication by Wu and colleagues (*23*). Among these, we found that *lophotrochin* is expressed in ciliary band clusters of both oyster and flatworm larvae, while *trochin* is found only in ciliary band clusters of the oyster larva (fig. S16, A and B). Another eight spiralian-specific genes were identified in the ciliary band of the oyster, and the orthologs of four of these (named here *prototrochin*, *ciliarybandin*, *mullerin*, and *trochophorin*) are also expressed in flatworm ciliary band clusters (fig. S16). These genes have very low expression in adult planarian scRNA-seq (*49*) but are expressed at larval stages in the annelid *Owenia fusiformis* (*50*), suggesting that they may be specific to larval ciliary bands (fig. S16, C and D).

Among neuronal clusters, the strongest alignment was found between oyster apical neurons and flatworm *MIP^+^* neurons, both of which are found in the apical organs of the larvae. A total of 23 orthologous genes are coexpressed in these clusters and, among these, the gene *pde9* is also found in the apical region of the larva of the annelid worm *P. dumerilii* (*51*) (see fig. S17).

#### Comparison of lophotrochozoan larvae and echinoderm pluteus

We extended our comparisons to include data from the distantly related sea urchin pluteus larva (*18*). The only meaningful matches that we recovered between oyster trochophore and pluteus larval cell clusters were for ciliary cells and myocytes. Ciliary cells coexpressed a total of 116 oyster genes and 87 sea urchin genes. Among these, we found many that overlapped with those shared between flatworm and oyster (e.g., *annexins*, *calmodulins*, *calcineurins*, *centrins*, *cubulins*, *various dyneins*, *enkurin*, and *tektins*). We found two TFs that were coexpressed (*Sp-foxJ* and *Cg-foxA/Cg-foxL1*, and *Sp-pax9* and *Cg-pax2/5*); however, these appear to be paralogs and are not the same TFs as those shared between oyster and flatworm (for a full list, see fig. S18). SAMap analysis did not align oyster and sea urchin neurons nor pluteus skeletal cells to oyster shell gland cells.

The comparison between the Müller’s larva and the echinoderm pluteus showed alignment of myocytes, neurons, and gut cells. For pluteus neurons, the best alignment is with ciliary neurons of the flatworm larva; these share 20 flatworm and 16 sea urchin genes including several *neuronal acetylcholine receptors*, *calmodulins*, the gene *synaptogyrin*, and *delta*. *Delta* expression is found in the brains of several other animals and in the apical neurons of the oyster larva (*51*).

We also find a match between the larval guts of Müller’s and pluteus larvae with cells coexpressing 116 flatworm and 61 sea urchin genes including several *dehydrogenases*, *glutathione S-transferase*, *ATP synthetases*, *glutamyl aminopeptidases*, *peroxiredoxin-6*, *retinol dehydrogenases*, *sterol carriers*, *apolipoproteins*, and *heme binding proteins*. Pluteus and Müller’s larval guts also coexpress the *macrophage mannose recepto*r gene (ManrC1A), which is a stomach-specific marker of the sea urchin larval gut but do not coexpress any TFs (*52*).

Lastly, we observed a strong alignment (alignment score, ~0.8) between the exocrine pancreatic-like cell of the sea urchin and the gut-related cluster of the Müller’s larva. These clusters only coexpressed 19 flatworm genes and 17 urchin genes; however, many of these (e.g., *cpa2L*, *carboxypeptidase B*, *trypsin*, and *pancreatic lipase-related* and *ptf1a*) are very specific markers for the exocrine pancreatic-like cells of the sea urchin (*52*). Cells from these clusters are found in the upper gut in both larvae (although in the flatworm, they are also found elsewhere around the gut) (Fig. 4). Exocrine pancreatic-like cells have been described in adults of cephalochordates, tunicates, cnidarians, and in the Pacific oyster (*53*–*55*). Previously, they have only been described in larval stages of the sea urchin *Strongylocentrotus purpuratus* (*54*).

### General discussion

Ciliated larval stages are widespread across animal phyla, and their origins and evolution are long-standing problems in the field of evo-devo. Here, we present high-quality single-cell atlases for larvae of two phyla within the super clade Lophotrochozoa: the trochophore larva of the oyster and the Müller’s larva of the polyclad flatworm. We used these datasets to explore the cellular composition of the two larvae in the contexts of their unique biologies, evolutionary histories, and their potential homology. We find that, while the two larvae have putatively homologous cell families such as ciliary band cells, neurons, myocytes, and proliferative cells, each larva also presents its own idiosyncratic set of cells fitting it to its specific biological niche.

The trochophore larva of the Pacific oyster, at the stage analyzed here, lacks several features that are commonly shared across lophotrochozoan larvae, including protonephridia, a fully differentiated gut and an apical tuft. Despite its canonical trochophore-like appearance, the oyster larva also features several likely mollusc-specific innovations including an unexpectedly large set of transcriptionally distinct muscle types and multiple cells involved in making the shell. The young TAIs previously found for larval stages of bivalves using bulk transcriptomics are strongly affected by these clade-specific characters, most obviously by the derived molluscan shell transcriptome, and we suggest that measures based on bulk transcriptomics are not a reliable indicator of the recent evolution of trochophores (*3*, *55*).

The Müller’s larva is considered by some to be a highly derived trochophore and by others to be a convergently evolved larval type (*3*, *56*). We found that it displays multiple classical larval characters missing in the oyster trochophore, such as a large apical organ, paired protonephridia, and a complex gut.

To test the homology of shared cell types, we used SAMap to align our two datasets to each other and to the pluteus larva of the sea urchin. We found clear overlaps in orthologous gene expression at the level of cell family for myocytes, proliferative cells, and neurons. These cell families are likely to be homologous across adult Metazoa and cannot be used to demonstrate larval homology (*15*). We also identified potentially homologous cell types specific to larvae, including ciliary band cells and a subset of apical neurons.

Ciliary band cells of the oyster and flatworm larvae share over a hundred orthologous genes and, although many of these play a role in the ciliary apparatus, we find that they are not shared with other ciliated cell types such as protonephridia and pharyngeal cells. Moreover, ciliary bands of flatworm and oyster share several TFs (namely, *dbx*, *gsc*, *otx*, *pbx*, *zfhx*, and *pax6*) and five spiralian-specific genes that appear to be larval specific (including *lophotrochin*) (*7*, *10*, *23*, *46*–*48*, *56*). *Lophotrochin* and *otx* are also found in ciliary bands of several other lophotrochozoan and bilaterian larvae (*7*, *10*, *23*, *46*–*48*, *57*). When broadening the comparison to the sea urchin larval ciliary bands, we still recover around 100 coexpressed genes between oyster and sea urchin, although we find no TFs among these genes. We also do not recover a match (alignment score, >0.2) with ciliary band cells of the flatworm larva.

Our data also support the potential homology of a subset of neurons located in the apical organ of both the oyster trochophore larva and the Müller larva of the flatworm. These apical neurons coexpress several orthologous genes, including the gene *pde9* also found in the apical organ of the trochophore larva of the annelid *P. dumerilii* (*51*). In the flatworm (but not in the oyster), these neurons also express the protostome-specific proneuropeptide *MIP*, whose expression is reminiscent of that observed in the larvae of the two annelids *P. dumerilii* and *Capitella* teleta** (*42*).

When comparing neurons with the sea urchin larva, we find several TFs (namely, *awh*, *hbn*, *delta*, and *zic*) expressed in both sea urchin and oyster apical neurons (*18*, *26*). These TFs are commonly shared across neurons of adult animals and do not represent a strong indication of larval homology (*26*). Sea urchin neurons do not match MIP^+^ cells of the flatworm but rather to the ciliary-motor neurons, with which they share the expression of 20 genes including *delta*. While equivalent cells are not present in the mollusc trochophore, these ciliary motor-neurons have nevertheless been described in other canonical trochophores such as that of the annelid *Platynereis* (*6*, *7*, *57*, *58*).

Overall, the most persuasive evidence that we present here for homology of Lophotrochozoan larvae is in the similarities in transcriptional signatures that we recovered in ciliary band cells and apical neurons. We believe that these, together with morphological and developmental similarities previously shown (*6*, *7*, *58*, *59*), point to the possible homology of at least trochophores and Müller’s larvae. Our comparison was, however, limited by the simplicity of the oyster trochophore larva nervous system and its lack of protonephridia and gut. Although these may develop later at the veliger stage, we chose to sample a typical trochophore larva as this has been proposed as ancestral (*1*, *3*, *9*, *60*). Sampling of different developmental stages, for both canonical trochophores and other larval types, should allow us to extend the comparison to other larval cells such as those forming the protonephridia and larval gut and may reveal stronger similarities.

The data presented here do not support the homology of lophotrochozoan larvae to deuterostome larvae although we recovered some interesting similarities between the larval gut of flatworm and sea urchin larvae and a clear match between the pluteus exocrine pancreatic-like cells and a cluster of gut related cells in the flatworm (*52*). This finding further confirms the presence of pancreatic-like cells in protostomes and is only the second occurrence described in ciliated larvae (*53*).

## MATERIALS AND METHODS

### Animal husbandry

*C. gigas* individuals, raised in farms in Salcott Creek Essex, UK, were bought during the spawning season (May to August 2018 and 2019) from Richard Haward’s Oysters in Borough Market, London, UK. In the laboratory, oysters were kept at 16°C in running artificial seawater (ASW) and fed three times a week with Spirulina powder and invertebrate food supplement. For spawning, male and female *C. gigas* were shucked, and gametes were stripped and put in glass beakers containing artificial filtered seawater (AFSW). Eggs were left in AFSW for about 1 hour to improve synchronicity, and then a dilution of sperm was added. After 5 min, the water was tipped onto a 20-μm filter mesh, and fertilized eggs were washed several times to avoid polyspermy. Fertilized eggs were then collected from the mesh and placed in beakers of AFSW at either 20° or 25°C in an incubator. Trochophore larvae were collected on a 20-μm mesh after 16 hours (25°C) or 20 hours (20°C) before they grew paired shells at the veliger stage. We wanted to sample larvae at the trochophore stage as this has been homologized to the trochophore larva of annelids (*1*).

*P. crozieri* adult specimens were collected in coastal mangrove areas in the Lower Florida Keys, USA, in October 2019 using a Florida Fish and Wildlife Conservation Commission saltwater fishing license. Animals were brought to the United Kingdom under the import of invertebrate pet policy. In the laboratory, animals were kept at room temperature (RT) (~21°C) in plastic boxes filled with ASW. The water was changed daily for the first 2 weeks and then once every 2 to 3 days. The animals cannot be fed in the laboratory, and so they were kept starved. Whenever eggs were found, they were placed in separate containers and daily checked for hatchlings. Hatched larvae were transferred into a filter and washed several times in filtered seawater and then relaxed in 7.14% MgCl_2_•6H_2_O.

### Cell dissociation

*C. gigas* trochophore larvae were collected on a 20-μm filter mesh and transferred to low binding tubes. Larvae were washed several times in Ca2^+^ Mg2^+^-free ASW with the aid of a centrifuge. Animals were then moved into a 4-by-4 well and were left in the solution for 3 to 5 min. After this time, 300 μl of 0.5% Pronase (Roche, catalog number 10165921001) and 1% sodium thioglycolate (Sigma-Aldrich, T0632) in low-Ca2^+^ Mg2^+^-free ASW seawater were added, and the solution was gently pipetted up and down to mix. After 3 min, 10 μl of Liberase (5 mg/ml; Roche, catalog number 05401119001) was added. The solution was mixed by gently pipetting up and down, and then larvae were very gently manually triturated. The whole procedure never lasted more than 20 min to minimize cell mortality. One-day old larvae of *P. crozieri* were collected in a 40-μm filter and washed several times with filtered ASW. After cleaning, the larvae were washed several times with Ca2^+^ Mg2^+^-free ASW to prepare for dissociation. Larvae were collected in the center of the mesh and transferred to a plastic cell culture petri dish, and most of the Ca2^+^ Mg2^+ ^freeASW was removed by pipetting. Three hundred microliters of 1:100 solution of Prot14 (3.5 U/mg; Sigma-Aldrich, P5147) in low-Ca2^+^ Mg2^+^-free ASW previously activated at 37°C for 1 hour was added. The solution was pipetted gently for 5 to 10 min until most of the larvae were dissociated. After this time, the larval gut (which remained undissociated) was collected with the pipette and gently triturated until most cells separated and these were then added to the remaining cells. The dissociation process usually lasted around 15 min. For both animals, after dissociation, cells were washed several times in Ca2^+^ Mg2^+^-free ASW and checked for viability using fluorescein diacetate (e.g., Sigma-Aldrich, F7378) and propidium iodide (e.g., Sigma-Aldrich, P4170), and only samples with over 80% of viable cells were used for downstream capture.

### scRNA-seq data analysis

About 30,000 cells per sample were loaded into 10x chips following the manual instructions using the 10x Chromium controller and Chromium single-cell 3′ Kit v2, v3, or v3.1 (catalog number 120237, 10x Genomics, USA). Four single-cell libraries per animal were sequenced on Illumina NextSeq500 using a 2 × 75 paired-end kit to assess quality with shallow sequencing. Oyster reads were mapped against the chromosome scale genome of *C. gigas* generated by Peñaloza and colleagues (*60*). Flatworm reads were mapped onto the *P. crozieri* genome generated by our group (*61*). After shallow sequencing, output matrices were analyzed in Seurat v4.0.1 R according to the Seurat scRNA-seq R package documentation (*62*) (details of the following analysis can be found in the Zenodo folder). Cells with fewer than 200 genes, more than 20% of mitochondrial genes, and more than 5000 UMI were discarded. For the flatworm, the mitochondrial cutoff was set to 30%. At this point, we selected the best-quality libraries from each animal for downstream analysis: library Cg1 for the oyster and Pc3 and Pc4 for the flatworm (fig. S2 for the oyster and fig. S10 for flatworm).

From these libraries, we recovered 9314 cells for the oyster and 17,605 for the flatworm. We then normalized the datasets by dividing gene counts for each cell by the total counts for that cell and multiplying by 10,000 and then natural log-transformed using log1p. Subsequently, the top 2000 variable genes were found using the vst method, datasets were scaled, and principal components analysis was performed. Shared nearest neighbor graph was computed with 70 dimensions and initial resolution of 10, and clusters were then combined using a script for merging similar clusters from Musser *et al.* (*63*). Briefly, we calculated the average expression profiles for each cluster, and then normalized expression vectors were used to calculate Pearson correlations between each pairwise cluster combination. Their correlation was ranked from highest to lowest, and then we used a Wilcoxon rank sum test to calculate the number of differentially expressed genes between each pair. We merged all cluster pairs that differed by less than 20 differentially expressed genes with fold change. This process was performed iteratively on all cluster pairs and gave us 39 genetically distinct clusters in the oyster larva and 51 in the flatworm larva. To confirm that by only selecting the highest quality libraries we had not missed any cluster, we also similarly analyzed all libraries per species together. Oyster libraries had to be integrated as described in (*62*). In this case, we used a final resolution of 1, and the results from this analysis can be seen in figs. S1 and S5.

### Whole-mount ISH/HCR

Whole-embryo chromogenic ISHs for *C. gigas* were carried out as previously described (*64*) in 96-well “U”-bottom plate. Images were taken using a Zeiss Axio Imager M1. Probes for HCR were designed using the probe generator devised by Ryan Null (https://github.com/rwnull/insitu_probe_generator; and https://doi.org/10.5281/zenodo.4694867, v3.0.2) and then ordered from Integrated DNA Technologies, and amplifiers and solutions were bought from Molecular Instruments. Samples were rehydrated into PTW-DEPC (0.1% Tween 20 in 1× phosphate-buffered saline) and prehybridized in 200 μl of hybridization buffer for 30 min at 37°C, and then 50 μl of hybridization buffer with 8 nM each of probe was added. Samples were incubated overnight at 37°C in a thermoblock shaking at 750 rpm. The following day, samples were washed four times in 0.5 ml of HCR probe wash solution for 10 min at 37°C and then three times in 1 ml of 5XSSCT(DEPC) for 5 min at RT. Samples were preamplified in 100 μl of amplification buffer for 30 min at RT. At this point, 2 μl of each hairpin (for three probe experiments B1-H1, B1-H2, B2-H1, B2-H2, B3-H1, and B3-H2) per experiment was placed in different polymerase chain reaction (PCR) tubes, heated in a PCR thermocycler for 1 min and 30 s at 95°C, quickly spun down, and let it cool at RT for 30 min in the dark. Then, all hairpins were pooled in one tube with 50 μL per experiment of amplification buffer. Then, 50 μl of amplification buffer and hairpin mix was added to all tubes with the samples (final concentration of 40 nM hairpin). Samples were incubated overnight at 25°C in a thermomixer shaking at 750 rpm. The following day, samples were washed three times in 1 ml of 5X SSCT for 10 min at RT and then stained with 4′,6-diamidino-2-phenylindole (final concentration of 5 μg/ml) in 500 μl of PTW for 15 min. They were washed again twice in 500 μl of PTW and then transferred to 2,2′-thiodiethanol for imaging. The amplifiers used for the HCR experiments were B1-647, B2-594 and B3-488, and they were imaged using a Zeiss LSM-800 confocal microscope.

### Phylostratigraphy analysis

Gene age was estimated for both species using the GenERA tool (*65*). Briefly, the tool implements genomic phylostratigraphy (*66*) by searching for homologs throughout the entire National Center for Biotechnology Information (NCBI) non-redundant nucleotide database and combining these results with the NCBI Taxonomy to assign an origin to each gene and gene family in a query species. Once we assigned each gene to its phylostratum, we used the package myTAI (https://academic.oup.com/bioinformatics/article/34/9/1589/4772684) to calculate the TAI of the log-transformed gene average expression per cluster, calculated using theAverageExpression function of Seurat. Phylostrata enrichment analyses were computed using a hypergeometric test applied to the number of marker genes in each cluster belonging to each phylostrata compared to the global set of expressed genes.

### Cross-species cell-type comparison

SAMap v1.0.2 was used to compare the *C. gigas* and *P. crozieri* scRNA-seq datasets to each other, to the larval scRNA-seq dataset of *S. purpuratus* (*18*), and to the adult scRNA-seq dataset of *S. mediterranea* (*13*). The notebook with details of the analysis can be found in the Supplementary Materials.

### NP precursor search

NP precursors were identified by two different methodologies. First, BlastP analyses were performed using the previously published NP datasets with an *e* value of 1 × 10^–2^ (*58*, *67*–*69*). Then, predicted secretomes for *C. gigas* and *P. crozieri* were obtained using SignalP4.1 with the sensitive option (D-cutoff of 0.34). This secretome was used to search for precursors by regular expressions based on dibasic and monobasic cleavage sites as described before (*69*). These two methodologies produced a large database that included hundreds of hits that were manually curated by comparing with the NP precursor complement from several lophotrochozoan species (see table S1) (*58*, *69*).

## Supplementary Materials

### This PDF file includes:

Figs. S1 to S18 Table S1 References
