# Supplementary material for: Single-cell atlases of two lophotrochozoan larvae highlight their complex evolutionary histories
Source: Sci Adv. 2023 Aug 2;9(31):eadg6034. doi: 10.1126/sciadv.adg6034 (PMC10396302; doi:10.1126/sciadv.adg6034)
Supplement: Supplementary file 1 — Figs. S1 to S18 Table S1 References [file sciadv.adg6034_sm.pdf]

Science Advances
AAAS

Supplementary Materials for

Single-cell atlases of two lophotrochozoan larvae highlight their complex evolutionary histories

Laura Piovani *et al.*

Corresponding author: Laura Piovani, l.piovani@ucl.ac.uk; Maximilian J. Telford, m.telford@ucl.ac.uk

Sci. Adv. **9**, eadg6034 (2023)
DOI: 10.1126/sciadv.adg6034

This PDF file includes:

Figs. S1 to S18
Table S1
References

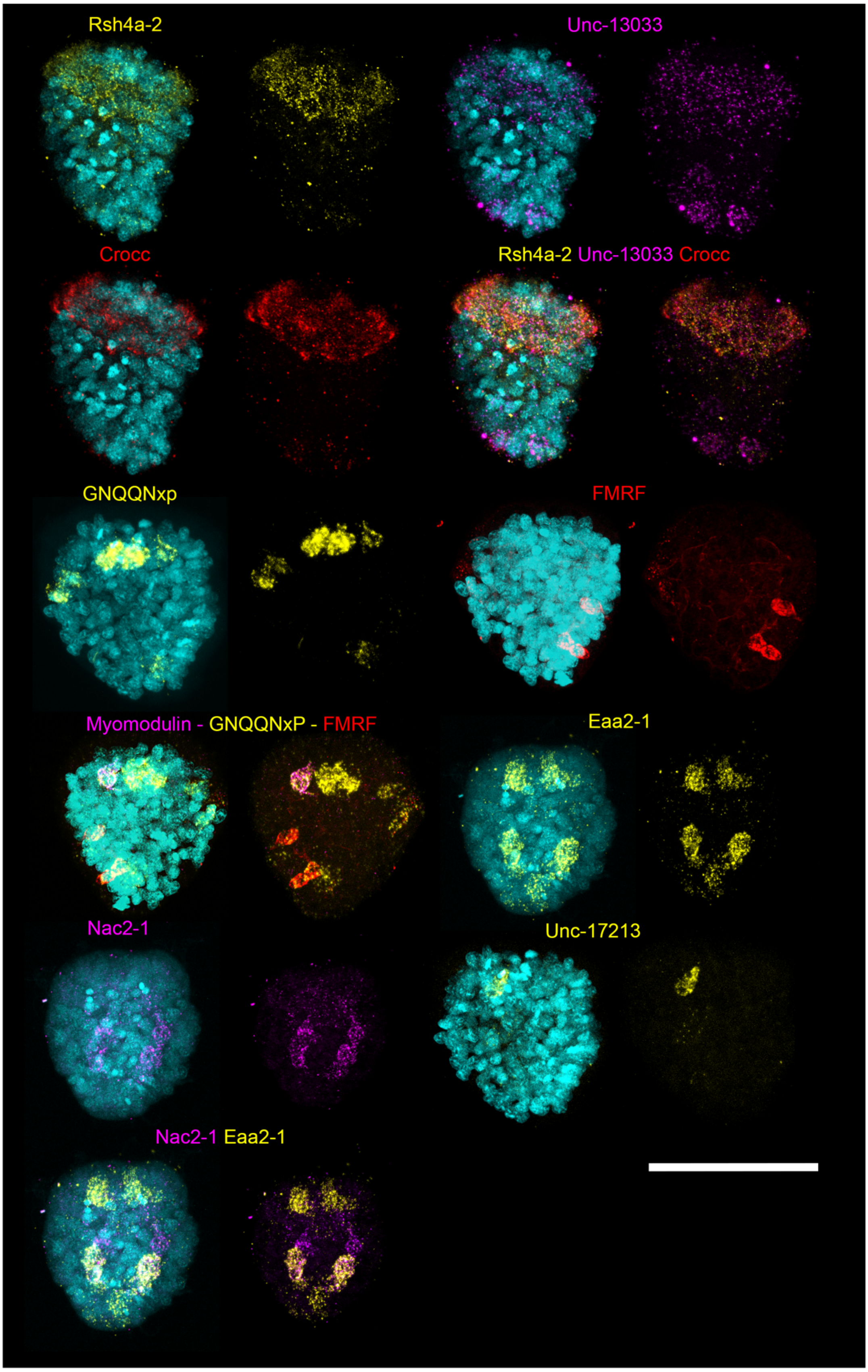

Fig. S1 HCR of marker genes for oyster larva scRNA-seq dataset. HCR expression of cluster markers shown in figure 2 with and without DAPI in a larger format. Scale bar is 50µm.

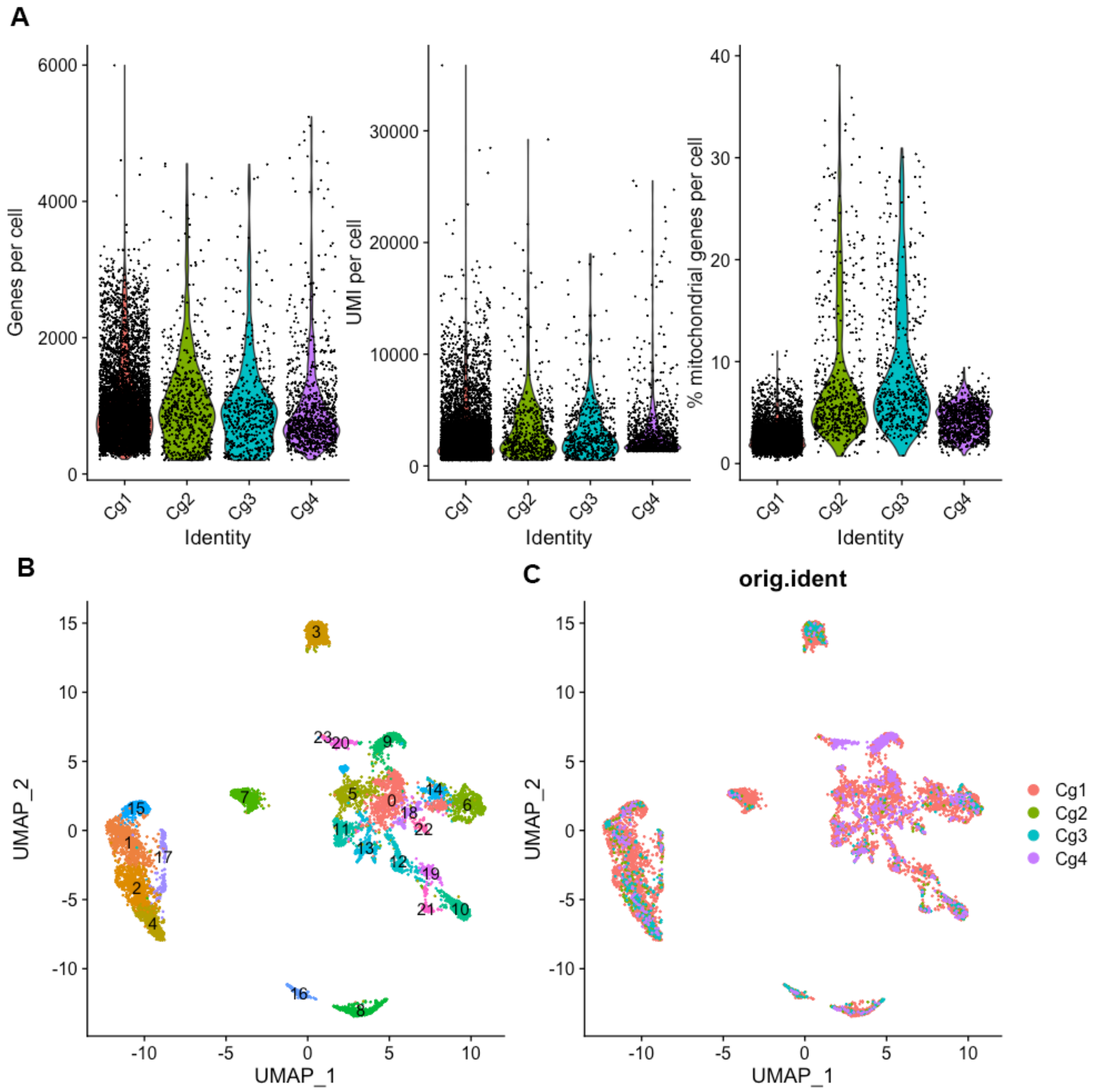

Fig. S2 Quality assessment of initial shallow sequencing of oyster trochophore scRNA libraries. A) Violin Plots showing gene number per cell (nFeature_RNA), UMI per cell (nCount_RNA) and percentage of mitochondrial genes (percent_mito) per cell in each sample (Cg1, Cg2, Cg3 and Cg4). Cg2 and Cg3 are technical replicates from the same dissociation. Sample Cg1 (used for downstream analysis) presents more overall cells, higher genes and UMIs and lower mitochondrial gene content. B) UMAP of integrated samples Cg1, Cg2, Cg3 and Cg4 coloured by cell clusters C) UMAP of integrated samples Cg1, Cg2, Cg3 and Cg4 coloured by sample of origin shows Cg1 cells are present in all clusters.

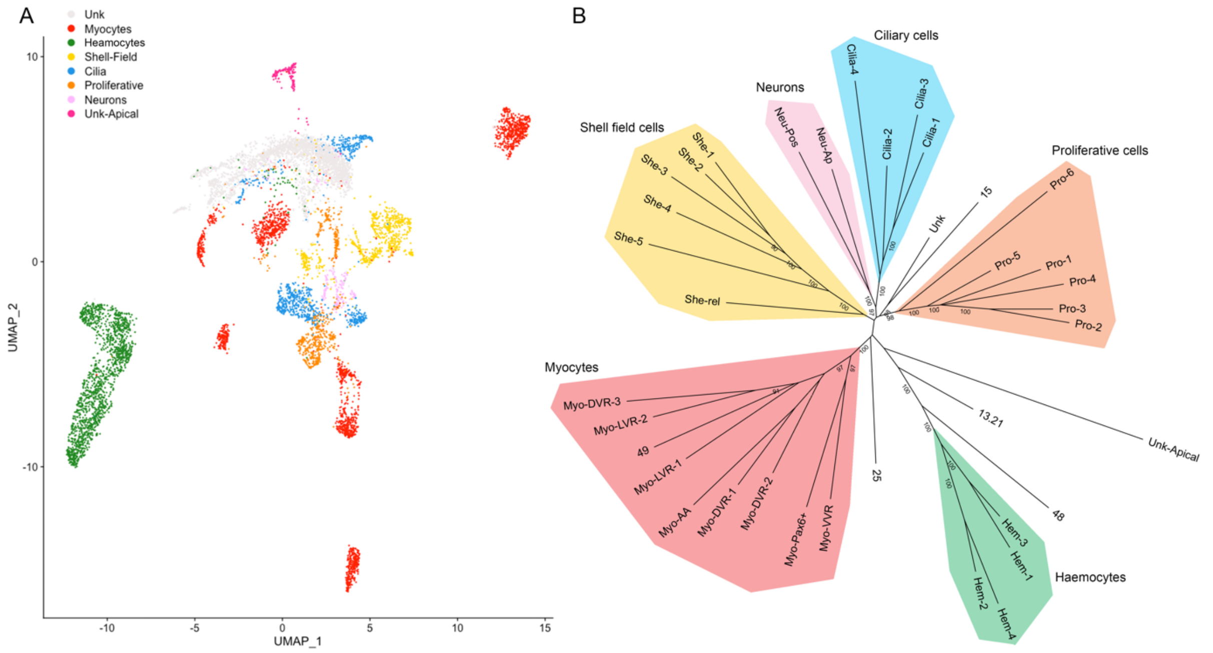

Fig. S3. Oyster trochophore larva cell type families. A) UMAP coloured by cell type family and B) Cell type tree showing bootstrap support >90. Cell type tree showing relationship between clusters calculated as Euclidean distance;
bootstrap values represent % of time that clade was recovered (10000 repeats). We recover 6 cell type families: ciliary cells, neurons, myocytes, shell field cells, proliferative cells and haemocytes. The oyster immune system starts developing at the trochophore stage (*70*) and we identified four clusters expressing haemocyte related genes including *thymosin-beta*, *flotillin-2* and the TF *tal-1* (*71–73*). ISH for haemocyte cluster markers (*irx3*, *lhx2-1 and tktl2-3*; Fig. 2B and 2C) show expression in two patches on either side of the developing gut, which appear to be connected anteriorly. We also identified four clusters that express proliferative markers such as *mago-nashi 2* (*mgn2*), *sumo3*, *pcna* and *CBX1* that play a role in stem cell proliferation (*74–77*). ISH for markers of these clusters (*a1cf-1* and *unchar-10915*) showed expression in the region of the developing gut. This is in line with the previous observation that, at the trochophore stage, the gut is still developing (*31*).

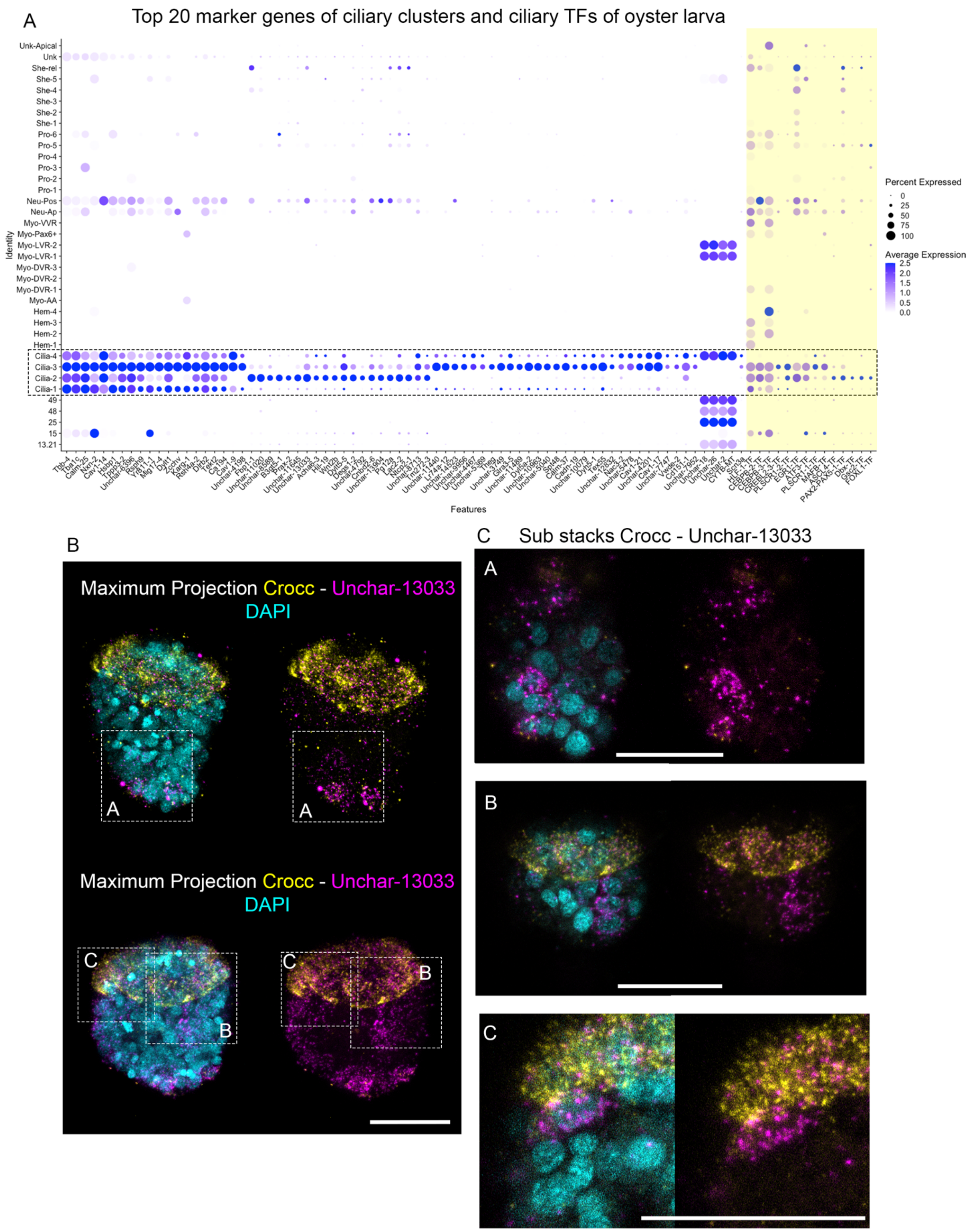
A
Top 20 marker genes of ciliary clusters and ciliary TFs of oyster larva
Unk-Apical
Unk
She-rel
She-5
She-4
She-3
She-2
She-1
Pro-6
Pro-5
Pro-4
Pro-3
Pro-2
Pro-1
Neu-Pos
Neu-Ap
Myo-VVR
Myo-Pax6+
Myo-LVR-2
Myo-LVR-1
Myo-DVR-3
Myo-DVR-2
Myo-DVR-1
Myo-AA
Hem-4
Hem-3
Hem-2
Hem-1
Cilia-4
Cilia-3
Cilia-2
Cilia-1
49
48
25
15
13.21
Identity
Features
Percent Expressed
0
25
50
75
100
Average Expression
2.5
2.0
1.5
1.0
0.5
0.0
B
Maximum Projection Crocc - Unchar-13033
DAPI
A
A
Maximum Projection Crocc - Unchar-13033
DAPI
C
B
C
B
C
Sub stacks Crocc - Unchar-13033
A
B
C

Fig. S4. Details of ciliary band markers in the oyster trochophore larva. A) Dotplot of top 20 marker genes for ciliary clusters and TFs in yellow. Dotplots show expression of genes (x axis) in each cell cluster (y axis) of the *C. gigas* scRNAseq, blue dots indicate average expression, size of dots indicate percentage of cells expressing the gene. Notice how cluster Cilia-1 shares all marker with other ciliary band clusters, Cilia-2 has unique markers, Cilia-3 share most markers with Cilia-4 but Cilia-4 present a subset shared with other clusters. B) Maximum projection of HCR for gene marker Crocc (Cilia-3 and Cilia-4) and Unchar-13033 (Cilia-2) and C) substacks of details show that Cilia-3 and Cilia-4 are prototrochal cells while Cilia-2 contains cells from the telotroch and a few cells posterior to the prototroch. Scale bars are 25 μm.

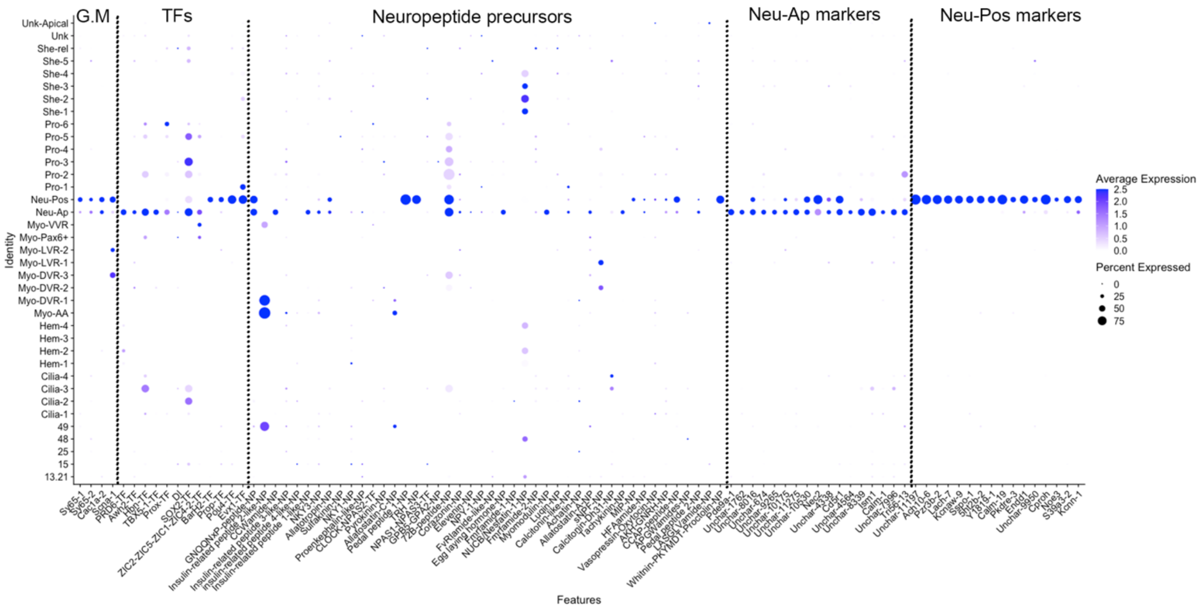

Fig. S5. Neuronal markers and neuropeptide precursors expression in the trochophore oyster larva. Dotplots show expression of genes (x axis) in each cell cluster (y axis) of the *C. gigas* scRNAseq, blue dots indicate average expression, size of dots indicate percentage of cells expressing the gene. Genes shown here general neuronal markers (G.M), TFs specific to neuronal clusters, neuropeptide precursors and top 20 apical and posterior neuronal markers.

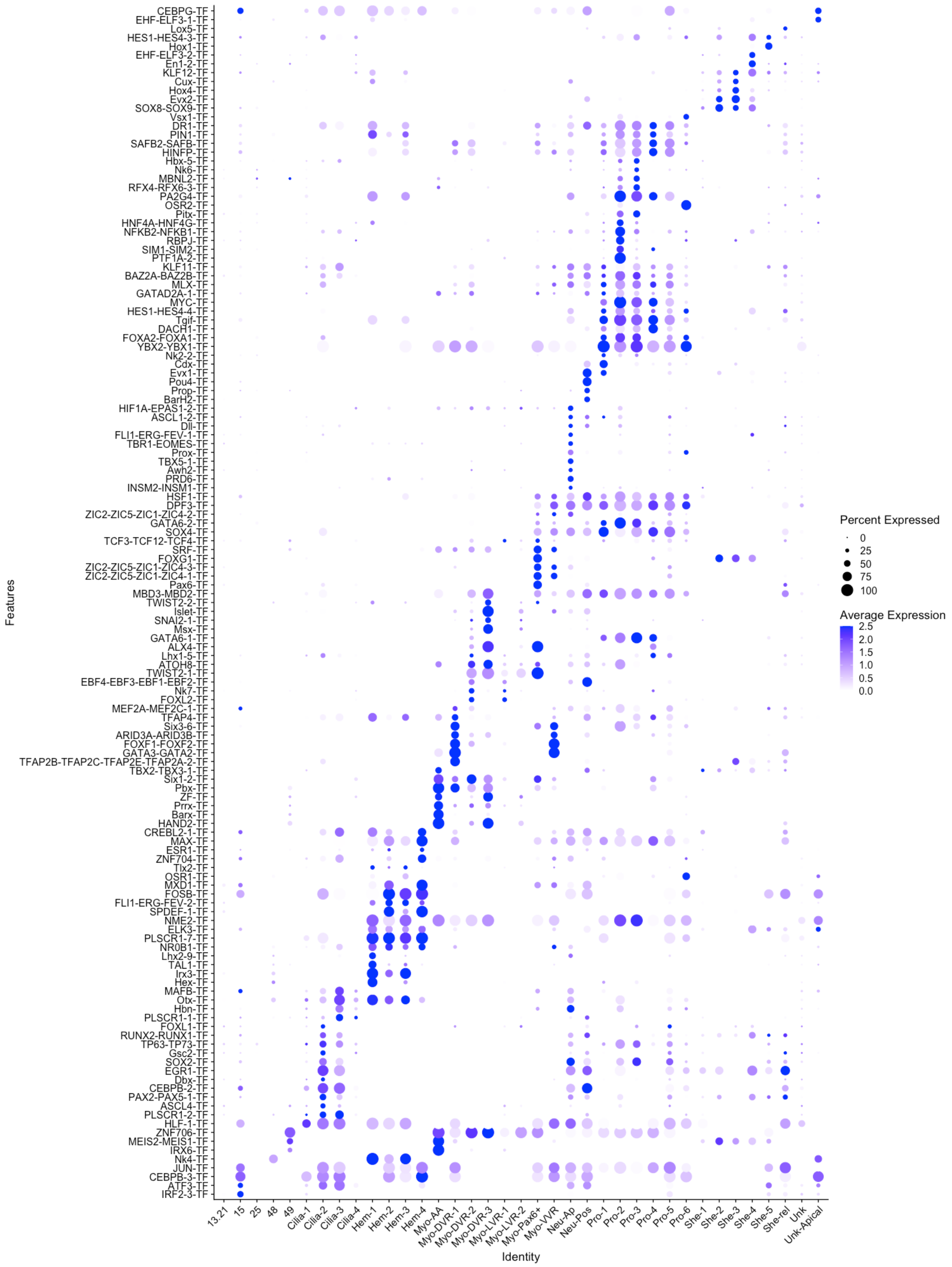

Fig. S6. Expression of TFs in the Oyster larva scRNA. Different myocytes clusters express different subsets of TFs. Dotplots show expression of genes (y axis) in each cell cluster (x axis) of the oyster scRNAseq, blue dots indicate average expression, size of dots indicate percentage of cells expressing the gene. Genes on the y axis are all TFs found in the oyster larva that are markers for a cluster.

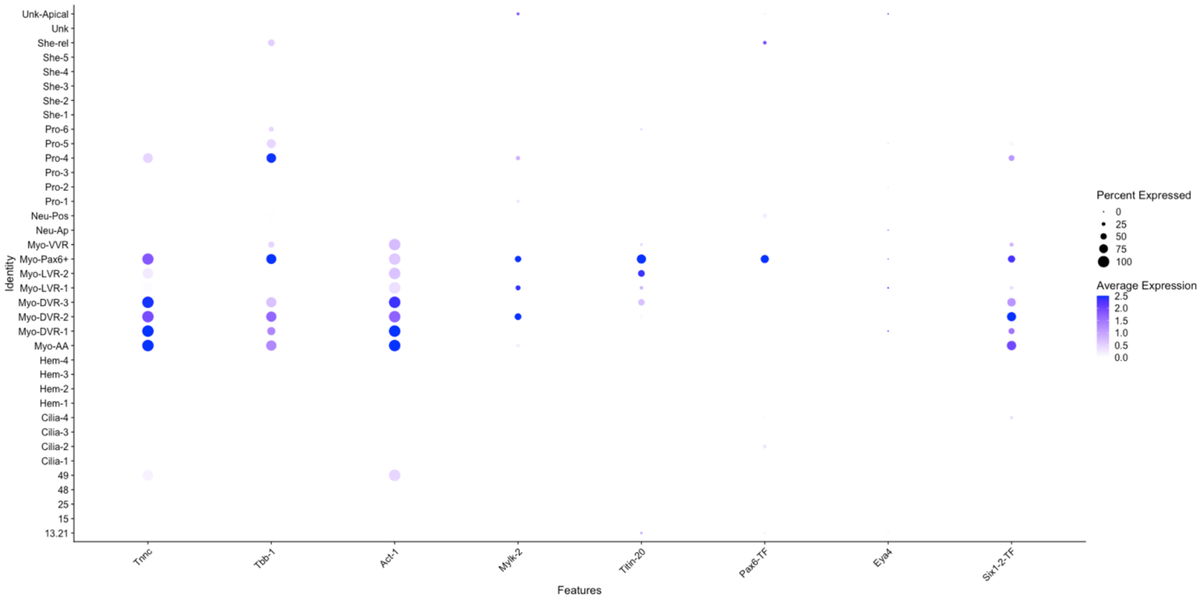

Fig. S7. Expression of myocyte markers and "eye master regulators" in the Pax6+ cluster of the oyster larva scRNA. Dotplots show expression of genes (x axis) in each cell cluster (y axis) of the oyster scRNAseq, blue dots indicate average expression, size of dots indicate percentage of cells expressing the gene.

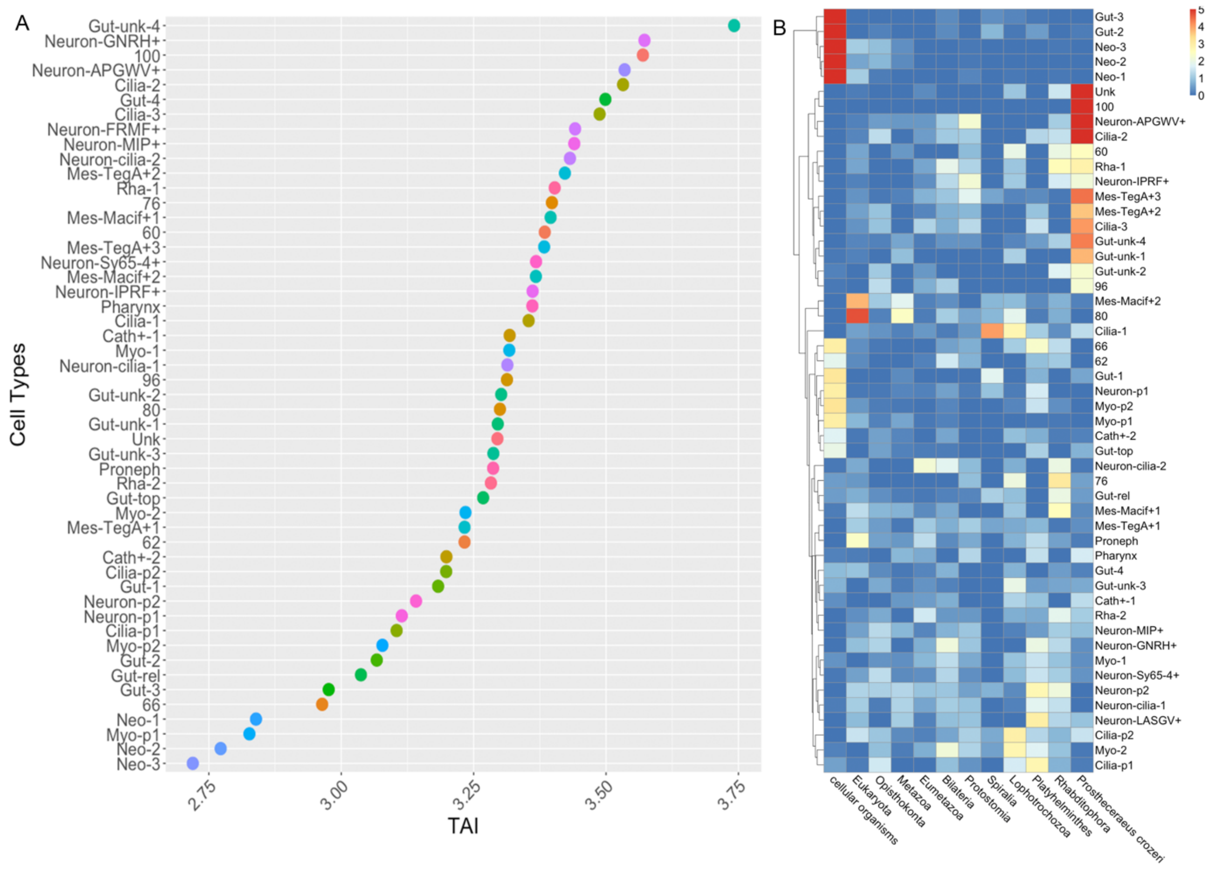

Fig. S8. Gene age analyses in different cell types of the flatworm larva. A) Transcriptome age indeces (TAI) for different cell types, smaller TAI values correspond to “older” gene age. Gene age is inferred using a phylostratigraphy approach, then transcriptomic age index is calculated on the log transformed gene average expression per cluster. B) Heatmap showing enrichment test −log10(P value) of enrichment test of for phylostratum of marker genes phylostrata per cell type in the flatworm. Enrichment was computed using a hypergeometric test applied to the number of marker genes in each cluster per phylostrata compared to the global set of expressed genes.

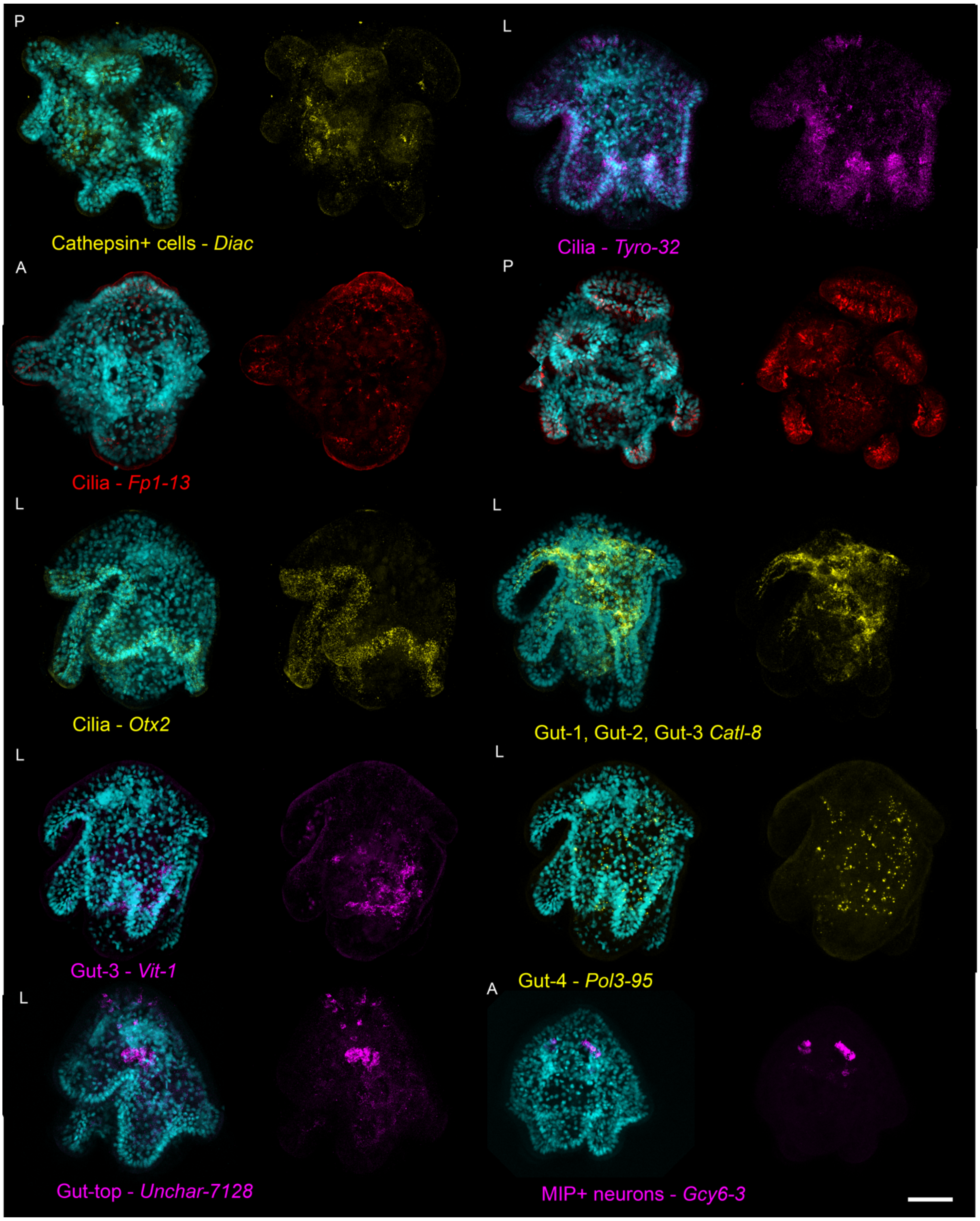
P
Cathepsin+ cells - Diac
L
Cilia - Tyro-32
A
Cilia - Fp1-13
P
L
Cilia - Otx2
L
Gut-1, Gut-2, Gut-3 Catl-8
L
Gut-3 - Vit-1
L
Gut-4 - Pol3-95
L
Gut-top - Unchar-7128
A
MIP+ neurons - Gcy6-3

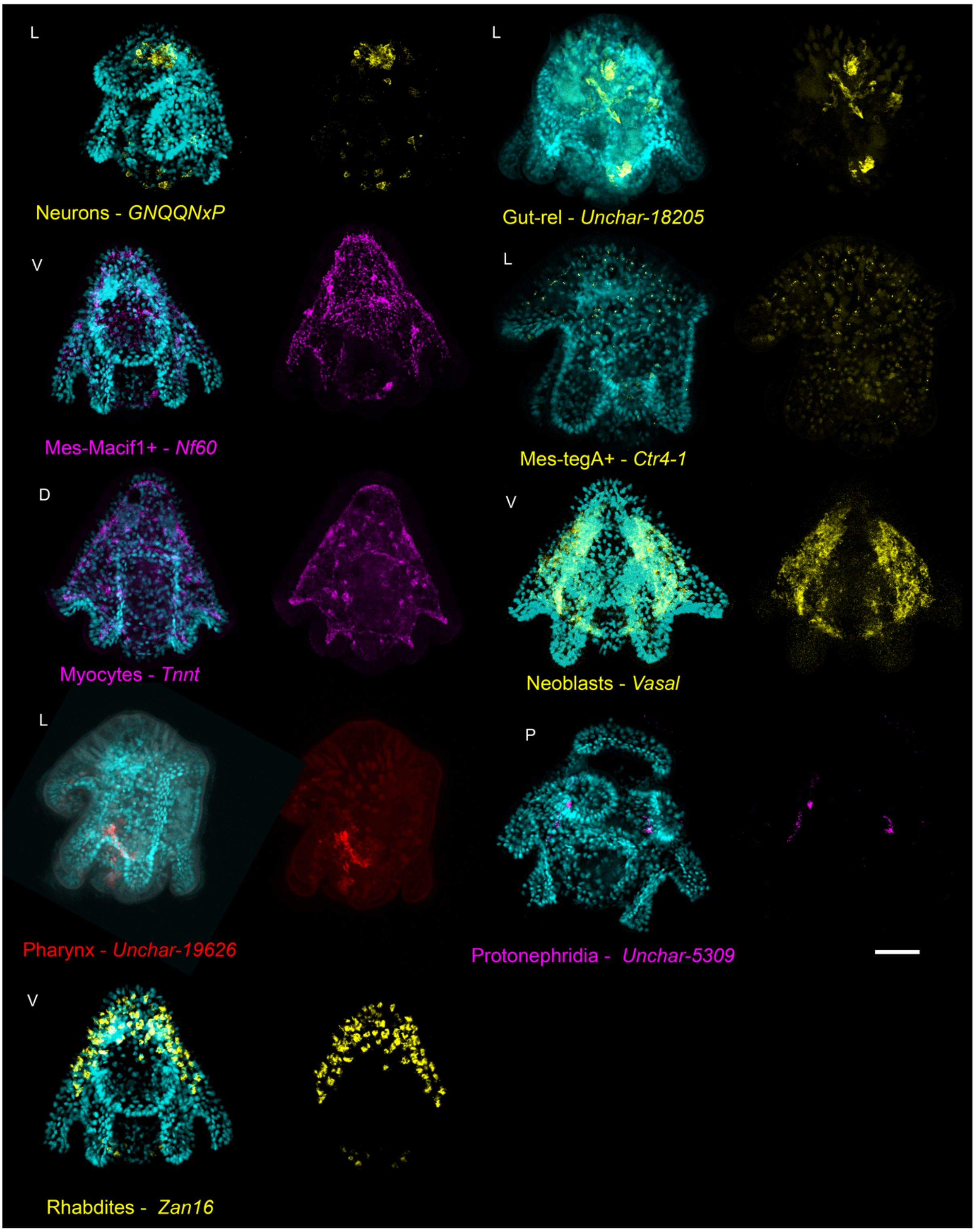

Fig. S9. Details of flatworm larva HCR from figure 4. HCR expression of cluster markers shown in figure 4 with and without DAPI in a larger format. A: apical view, P: posterior view, V: ventral view, D: dorsal view, L: lateral view with mouth on the left. Scale bars are 50 µm.

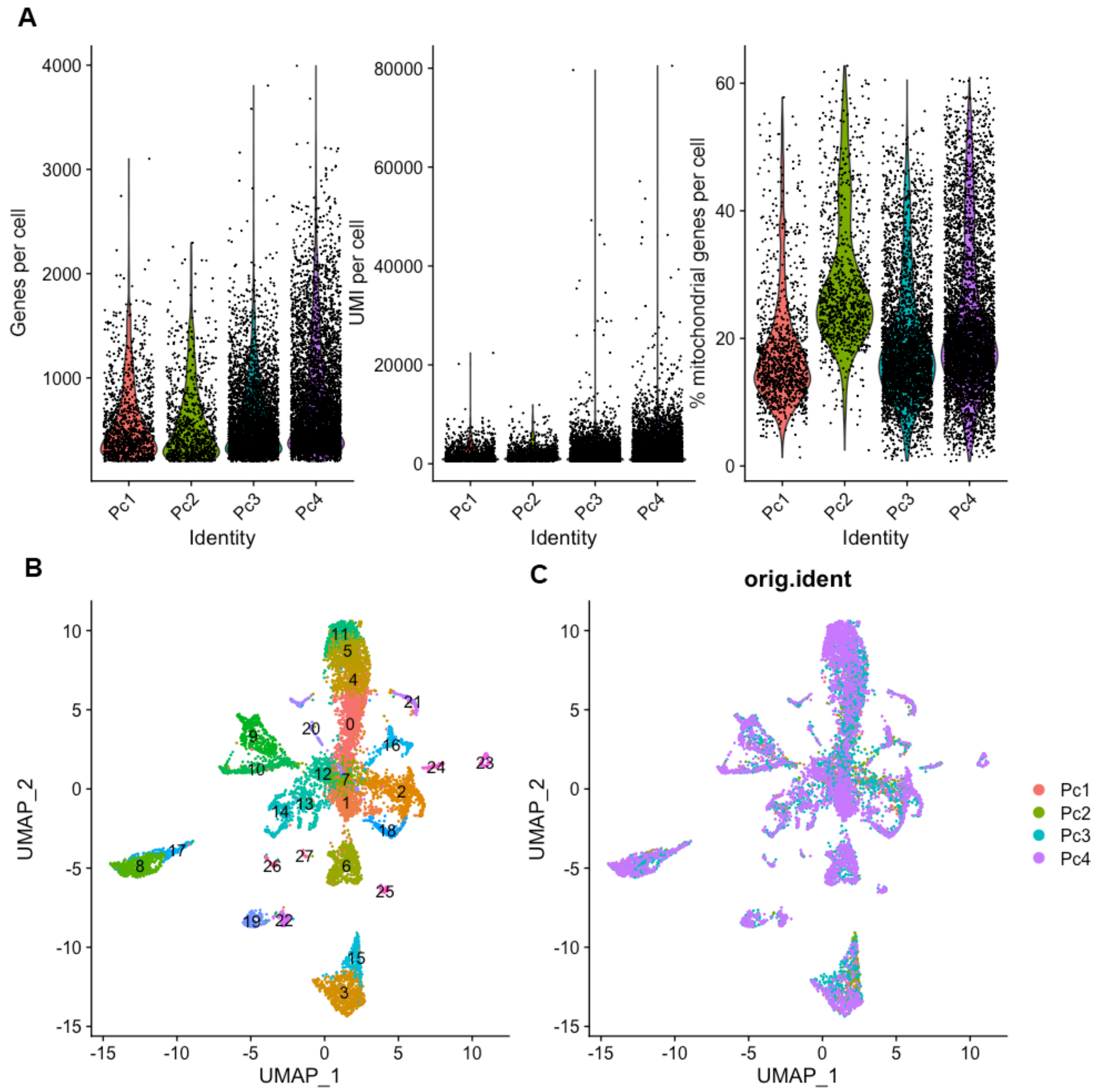

Fig. S10 Quality assessment of initial shallow sequencing of the flatworm Müller's larva scRNA libraries. A) Violin Plots showing gene number per cell (nFeature_RNA), UMI per cell (nCount_RNA) and percentage of mitochondrial genes (percent_mito) per cell in each sample (Pc1, Pc2, Pc3 and Pc4). Sample Pc3 and Pc4 (technical replicates used for downstream analysis) present more cells, higher genes and UMIs and lower mitochondrial gene content. Pc1 and Pc2 are technical replicates of each other from the same dissociation. B) UMAP of integrated samples Pc1, Pc2, Pc3 and Pc4 coloured by cell clusters C) UMAP of integrated samples Pc1, Pc2, Pc3 and Pc4 coloured by sample of origin shows that cells from Pc3 and Pc4 libraries are present in all clusters.

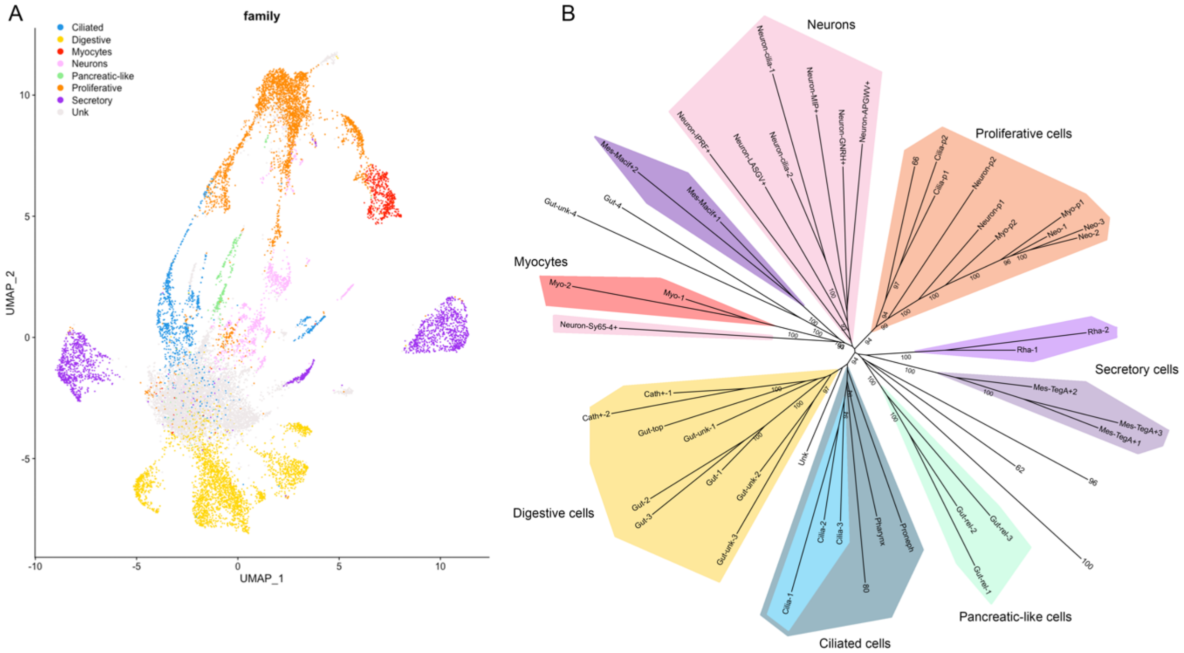

Fig. S11. Flatworm Müller's larva cell type families. A) UMAP coloured by cell type family and B) Cell type tree showing bootstrap support >90. Cell type tree showing relationship between clusters calculated as Euclidean distance;
bootstrap values represent % of time that clade was recovered (10000 repeats). We recover approximately 7 cell type families: ciliated cells, neurons, myocytes, secretory cells, proliferative cells, digestive cells and pancreatic-like cells.

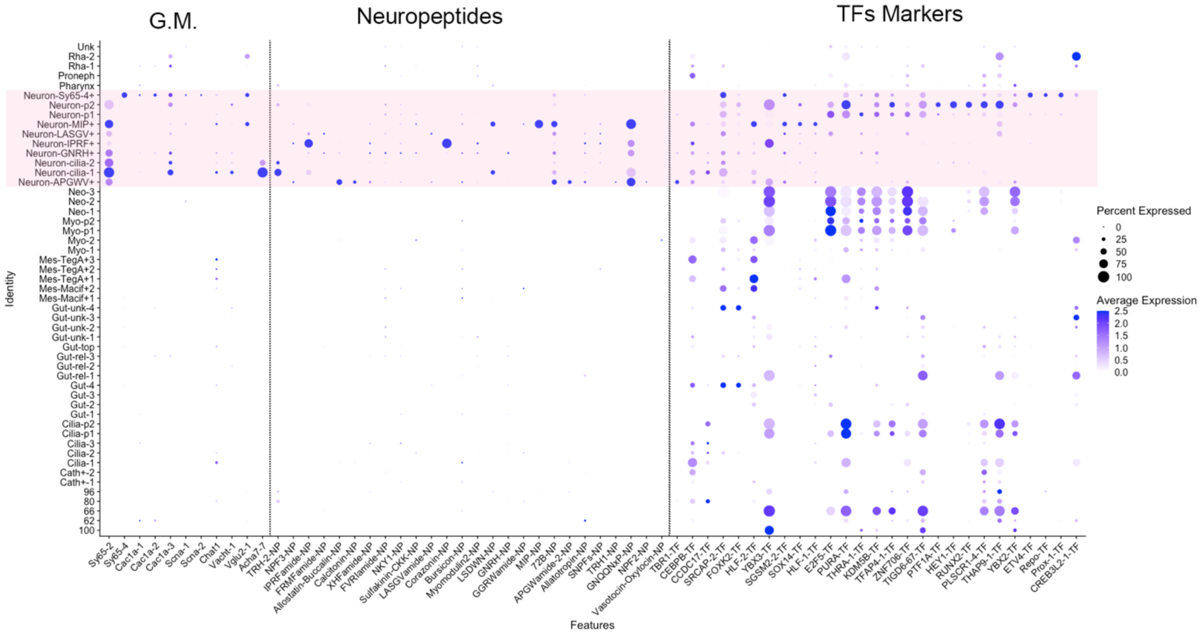

Fig. S12. Neuronal markers and neuropeptide precursors expression in the flatworm Müller's larva. Dotplots show expression of genes (x axis) in each cell cluster (y axis) of the *P. crozieri* scRNAseq, blue dots indicate average expression, size of dots indicate percentage of cells expressing the gene. Genes shown here general neuronal markers (G.M), TFs specific to neuronal clusters and neuropeptide precursors.

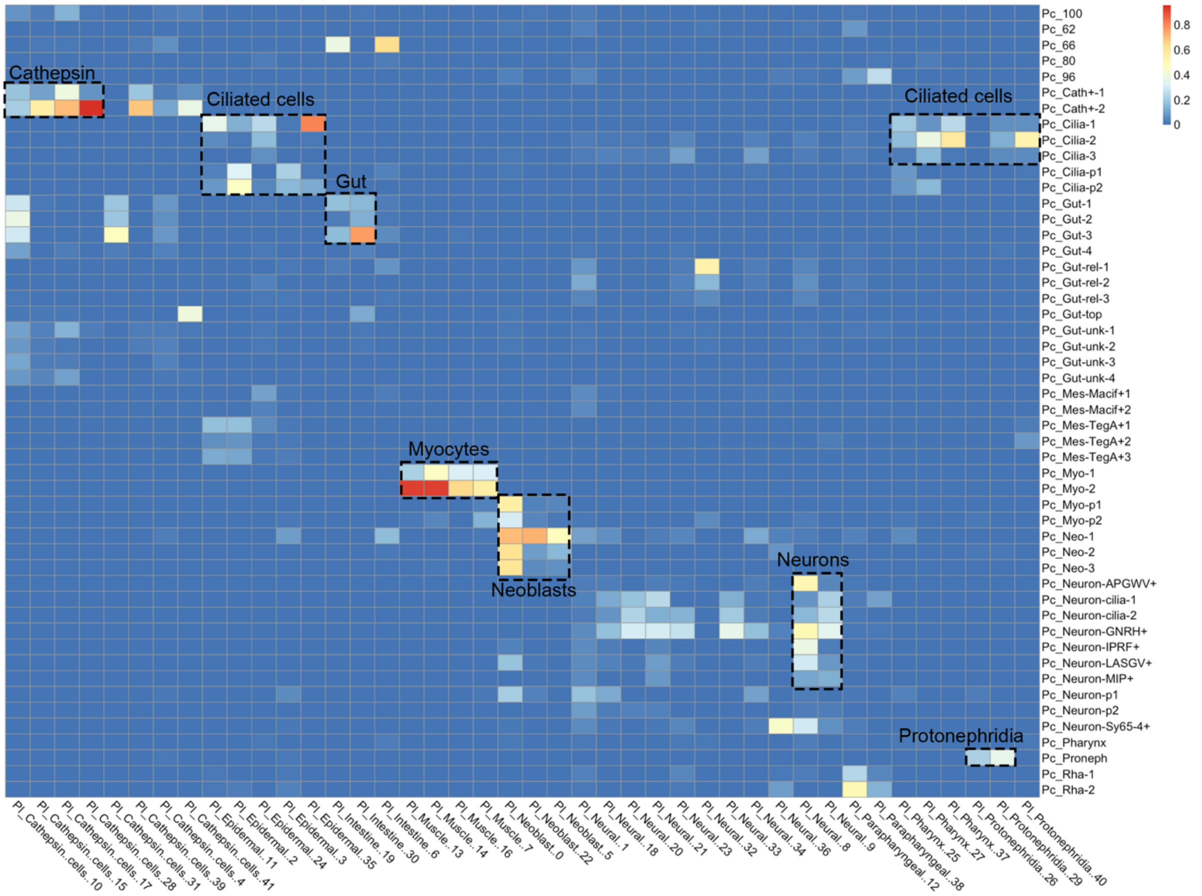

Fig. S13. SAMap alignment between *S. mediterranea* adult and *P.crozieri* larvae show similarities between neoblast, cathepsin cells, gut cells, myocytes, neurons and ciliated cells. *S. mediterranea* scRNAseq used here is from (13). SAMap alignment scores are defined as the average number of mutual nearest cross-species neighbors of each cell relative to the maximum possible number of neighbors (13). We find a match (SAMap alignment score >0.2) between putative *P.crozieri* neoblast cells and the known planarian neoblasts, suggesting that these are indeed neoblasts cells. We also found matches between muscle cells, several gut clusters, cathepsin cells, protonephridia, and several neuronal clusters. We found that ciliary band clusters of the Müller's larva match the epidermis, protonephridia, and pharynx clusters of the adult planarian worms (all of which are ciliated). However, adult planarian pharynx cells did not match with those of the larval polyclad.

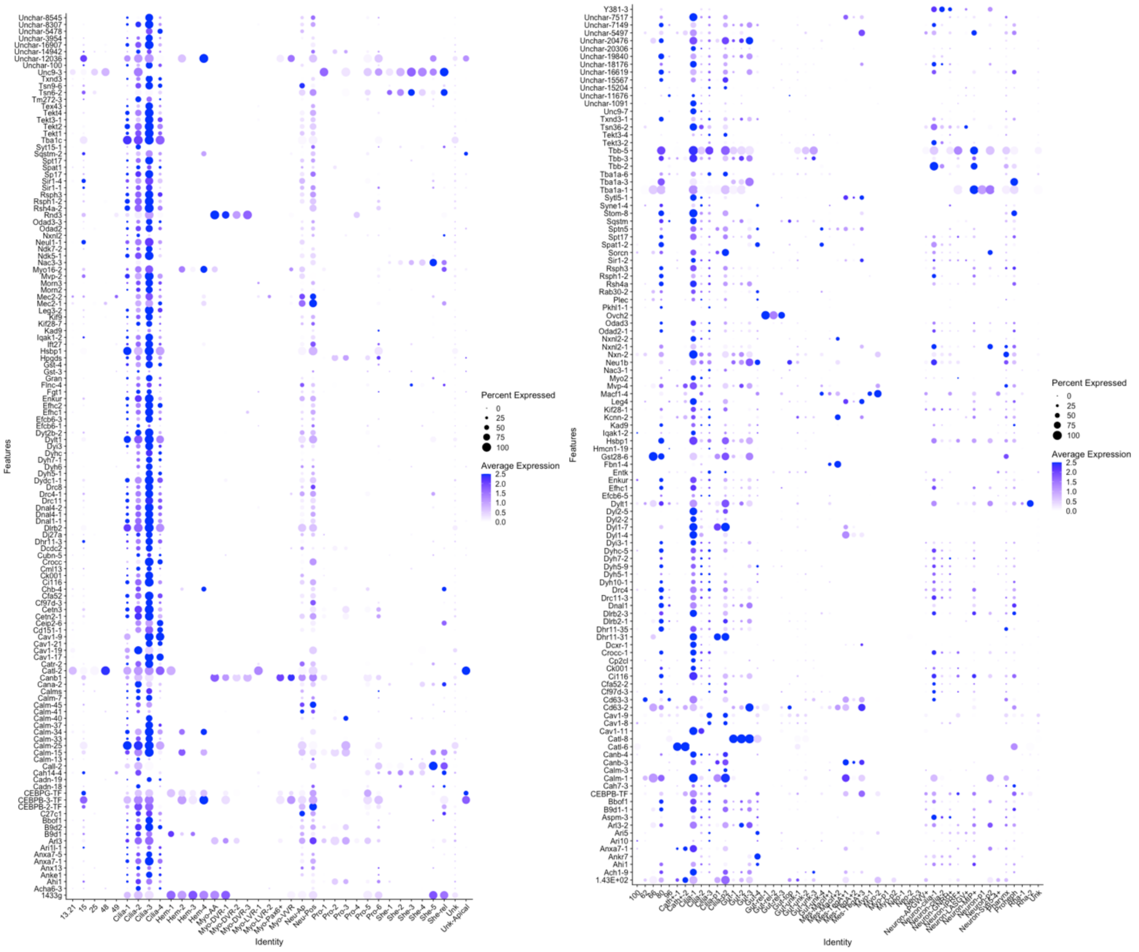

Fig. S14. Ciliary genes co-expressed between Oyster (left) and Flatworm (right) larvae generated with SAMap. SAMap calculates genes that are co-expressed between each aligned pair of cell types. Here we show the expression of co-expressed genes between the oyster (left) and the flatworm (right) ciliary clusters. Dotplots show expression of genes (y axis) in each cell cluster (x axis), blue dots indicate average expression, size of dots indicate percentage of cells expressing the gene. Notice how co-expressed ciliary band genes are not marking other ciliated cells in the flatworm larva such as protonephridia or pharyngeal cells.

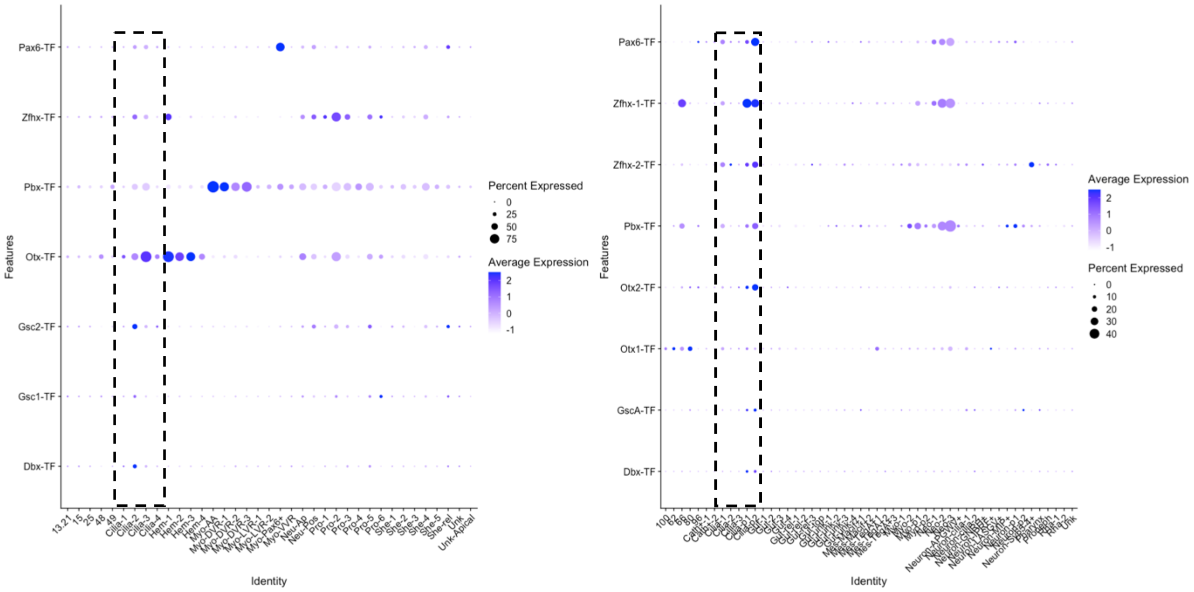

Fig. S15. Expression of homologous TFs in ciliary bands of the oyster (left) and flatworm (right). Dotplots show expression of genes (y axis) in each cell cluster (x axis) of the oyster scRNAseq (left) and flatworm (right), blue dots indicate average expression, size of dots indicate percentage of cells expressing the gene. Genes on the y axis are TFs expressed in the ciliary bands of both animals.

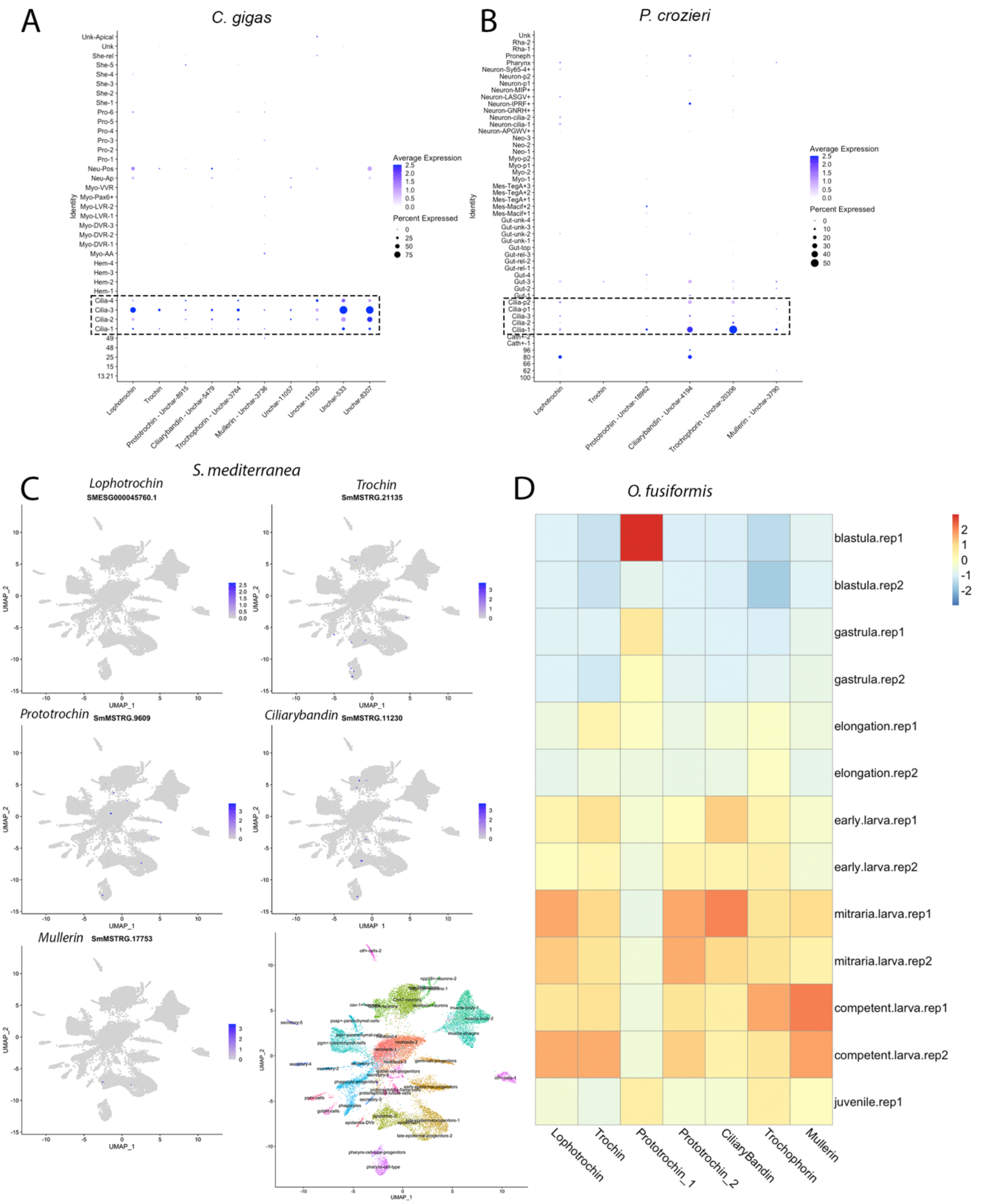

Fig. S16. Spiralian specific genes show expression in ciliary band of larvae but not adult nor juveniles. A-B) Expression of spiralian specific genes from a study by Wu and colleagues (23) in the A) oyster and B) flatworm larva ciliary band clusters. Dotplots show expression of genes (x axis) in each cell cluster (y axis), blue dots indicate average expression, size of dots indicate percentage of cells expressing the gene. Since four of these genes are expressed in ciliary bands of both larvae, we named them prototrochin, ciliarybandin, trochophorin and mullerin. C) Spiralian specific genes show very weak expression in planarian adult cells of *S.mediterranea* (49) but D) are expressed at larval stages in the annelid *O. fusiformis* (50).

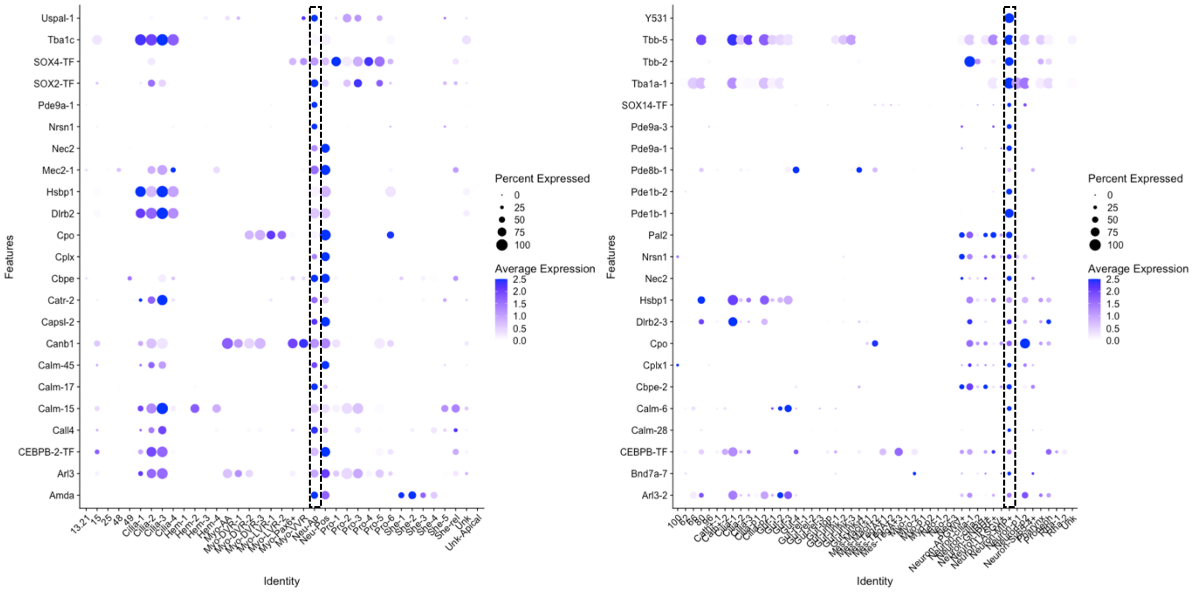

Fig. S17. Co-expressed genes between the apical neurons of the oyster (left) and the MIP+ neurons of the flatworm (right). SAMap calculates genes that are co-expressed between each aligned pair of cell types. Here we show the expression of genes co-expressed between the oyster apical neurons (left) and the flatworm MIP+ cells (right). Dotplots show expression of genes (x axis) in each cell cluster (y axis), blue dots indicate average expression, size of dots indicate percentage of cells expressing the gene.

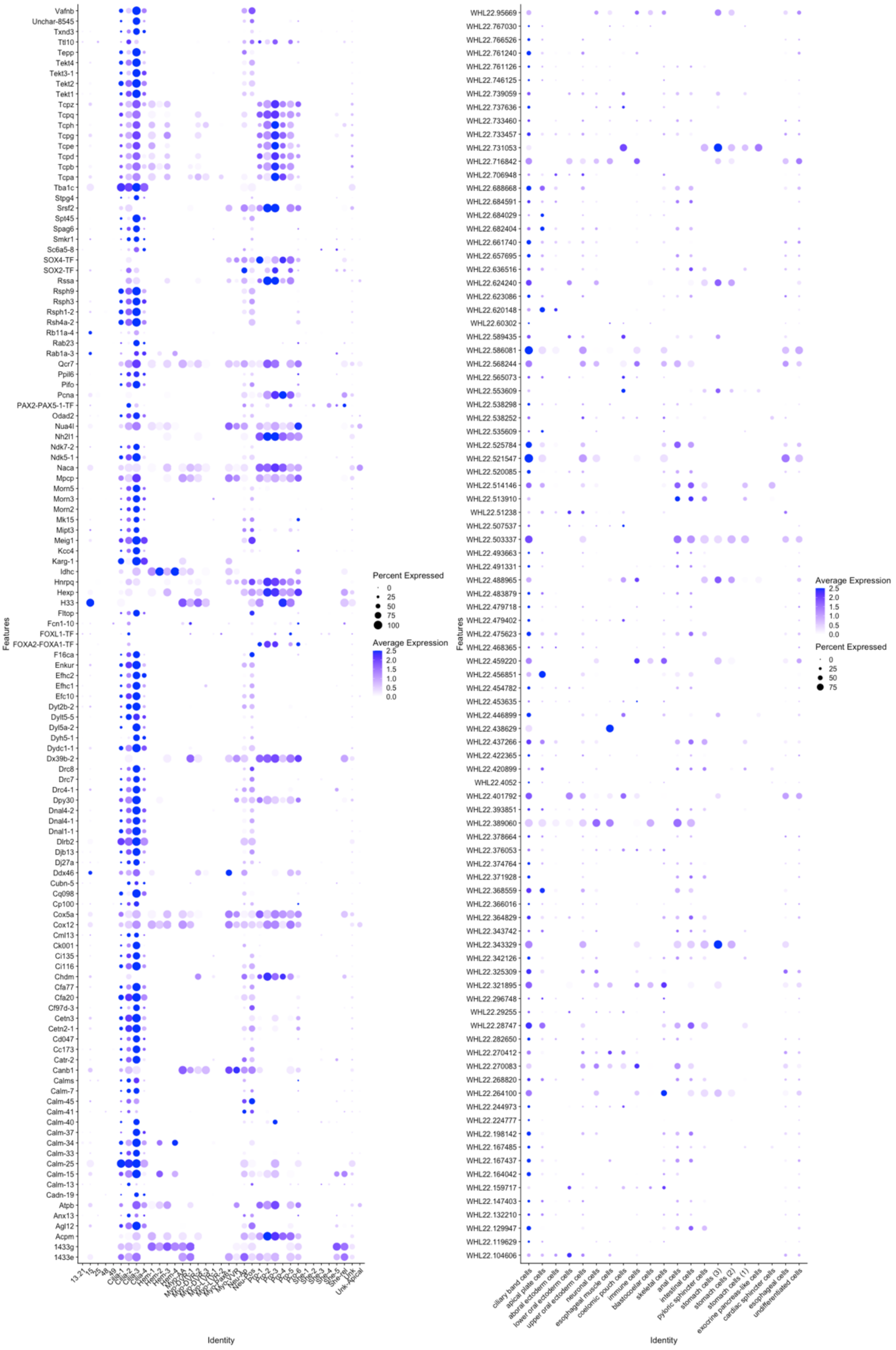

Fig. S18 Ciliary genes co-expressed between Oyster and Sea Urchin larva generated with SAMap. SAMap calculates genes that are co-expressed between each aligned pair of cell types. Here we show the expression of genes co-expressed between the oyster (left) and the sea urchin (right) ciliary clusters. Dotplots show expression of genes (y axis) in each cell cluster (x axis), blue dots indicate average expression, size of dots indicate percentage of cells expressing the gene.

Table S1. Presence (navy) and absence (white) of a set of Neuropeptides in Lophotrochozoa. The original data for brachiopods, nemertans, *P. dumerilii* and phoronids is from (*70*). Fasta files containing the sequence of all NP precursors for *C.gigas* and *P.crozieri* are present in supplementary materials on zenodo.

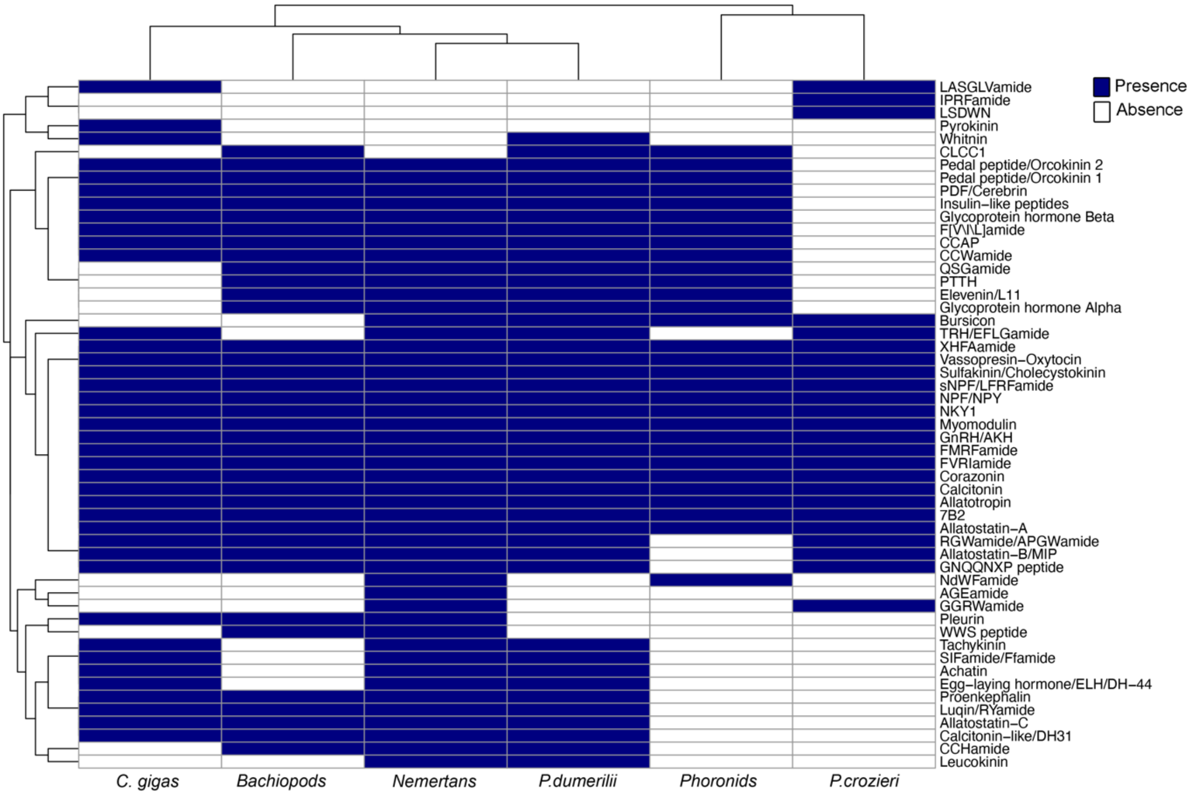

REFERENCES AND NOTES

1. C. Nielsen, Trochophora larvae: Cell-lineages, ciliary bands, and body regions. 1. Annelida and Mollusca. *J. Exp. Zool. B Mol. Dev. Evol.* **302**, 35–68 (2004).

2. A. Hejnol, M. Q. Martindale, J. Q. Henry, High-resolution fate map of the snail Crepidula fornicata: The origins of ciliary bands, nervous system, and muscular elements. *Dev. Biol.* **305**, 63–76 (2007).

3. C. Nielsen, Trochophora larvae: Cell-lineages, ciliary bands and body regions. 2. Other groups and general discussion. *J. Exp. Zool. B Mol. Dev. Evol.* **304**, 401–447 (2005).

4. K. A. Rawlinson, Embryonic and post-embryonic development of the polyclad flatworm Maritigrella crozieri; implications for the evolution of spiralian life history traits. *Front. Zool.* **7**, 12 (2010).

5. J. Wang, L. Zhang, S. Lian, Z. Qin, X. Zhu, X. Dai, Z. Huang, C. Ke, Z. Zhou, J. Wei, P. Liu, N. Hu, Q. Zeng, B. Dong, Y. Dong, D. Kong, Z. Zhang, S. Liu, Y. Xia, Y. Li, L. Zhao, Q. Xing, X. Huang, X. Hu, Z. Bao, S. Wang, Evolutionary transcriptomics of metazoan biphasic life cycle supports a single intercalation origin of metazoan larvae. *Nat. Ecol. Evol.* **4**, 725–736 (2020).

6. S. A. Maslakova, M. Q. Martindale, J. L. Norenburg, Vestigial prototroch in a basal nemertean, Carinoma tremaphoros (Nemertea; Palaeonemertea). *Evol. Dev.* **6**, 219–226 (2004).

7. H. Marlow, M. A. Tosches, R. Tomer, P. R. Steinmetz, A. Lauri, T. Larsson, D. Arendt, Larval body patterning and apical organs are conserved in animal evolution. *BMC Biol.* **12**, 7 (2014).

8. J. Girstmair, M. J. Telford, Reinvestigating the early embryogenesis in the flatworm *Maritigrella crozieri* highlights the unique spiral cleavage program found in polyclad flatworms. *Evodevo* **10**, 12 (2019).

9. G. W. Rouse, Trochophore concepts: Ciliary bands and the evolution of larvae in spiralian Metazoa. *Biol. J. Linn. Soc.* **66**, 411–464 (1999).

10. D. Arendt, U. Technau, J. Wittbrodt, Evolution of the bilaterian larval foregut. *Nature* **409**, 81–85 (2001).

11. A. Tanay, A. Sebé-Pedrós, Evolutionary cell type mapping with single-cell genomics. *Trends Genet.* **37**, 919–932 (2021).

12. D. Arendt, J. M. Musser, C. V. H. Baker, A. Bergman, C. Cepko, D. H. Erwin, M. Pavlicev, G. Schlosser, S. Widder, M. D. Laubichler, G. P. Wagner, The origin and evolution of cell types. *Nat. Rev. Genet.* **17**, 744–757 (2016).

13. A. J. Tarashansky, J. M. Musser, M. Khariton, P. Li, D. Arendt, S. R. Quake, B. Wang, Mapping single-cell atlases throughout Metazoa unravels cell type evolution. *eLife* **10**, e66747 (2021).

14. J. Li, J. Wang, P. Zhang, R. Wang, Y. Mei, Z. Sun, L. Fei, M. Jiang, L. Ma, W. E, H. Chen, X. Wang, Y. Fu, H. Wu, D. Liu, X. Wang, J. Li, Q. Guo, Y. Liao, C. Yu, D. Jia, J. Wu, S. He, H. Liu, J. Ma, K. Lei, J. Chen, X. Han, G. Guo, Deep learning of cross-species single-cell landscapes identifies conserved regulatory programs underlying cell types. *Nat. Genet.* **54**, 1711–1720 (2022).

15. A. Sebé-Pedrós, B. Saudemont, E. Chomsky, F. Plessier, M.-P. Mailhé, J. Renno, Y. Loe-Mie, A. Lifshitz, Z. Mukamel, S. Schmutz, S. Novault, P. R. H. Steinmetz, F. Spitz, A. Tanay, H. Marlow, Cnidarian cell type diversity and regulation revealed by whole-organism single-cell RNA-seq. *Cell* **173**, 1520–1534.e20 (2018).

16. J. A. Briggs, C. Weinreb, D. E. Wagner, S. Megason, L. Peshkin, M. W. Kirschner, A. M. Klein, The dynamics of gene expression in vertebrate embryogenesis at single-cell resolution. *Science* **360**, eaar5780 (2018).

17. H. M. T. Choi, M. Schwarzkopf, M. E. Fornace, A. Acharya, G. Artavanis, J. Stegmaier, A. Cunha, N. A. Pierce, Third-generation in situ hybridization chain reaction: Multiplexed, quantitative, sensitive, versatile, robust. *Development* **145**, dev165753 (2018).

18. P. Paganos, D. Voronov, J. M. Musser, D. Arendt, M. I. Arnone, Single-cell RNA sequencing of the *Strongylocentrotus purpuratus* larva reveals the blueprint of major cell types and nervous system of a non-chordate deuterostome. *eLife* **10**, e70416 (2021).

19. C. Peñaloza, A. P. Gutierrez, L. Eöry, S. Wang, X. Guo, A. L. Archibald, T. P. Bean, R. D. Houston, A chromosome-level genome assembly for the Pacific oyster *Crassostrea gigas*. *Gigascience* **10**, giab020 (2021).

20. L. Wu, K. E. Ferger, J. D. Lambert, Gene expression does not support the developmental hourglass model in three animals with spiralian development. *Mol. Biol. Evol.* **36**, 1373–1383 (2019).

21. F. Xu, T. Domazet-Lošo, D. Fan, T. L. Dunwell, L. Li, X. Fang, G. Zhang, High expression of new genes in trochophore enlightening the ontogeny and evolution of trochozoans. *Sci. Rep.* **6**, 34664 (2016).

22. F. Christodoulou, F. Raible, R. Tomer, O. Simakov, K. Trachana, S. Klaus, H. Snyman, G. J. Hannon, P. Bork, D. Arendt, Ancient animal microRNAs and the evolution of tissue identity. *Nature* **463**, 1084–1088 (2010).

23. L. Wu, L. S. Hiebert, M. Klann, Y. Passamaneck, B. R. Bastin, S. Q. Schneider, M. Q. Martindale, E. C. Seaver, S. A. Maslakova, J. D. Lambert, Genes with spiralian-specific protein motifs are expressed in spiralian ciliary bands. *Nat. Commun.* **11**, 4171 (2020).

24. S. Kakoi, K. Kin, K. Miyazaki, H. Wada, Early development of the Japanese spiny oyster (*Saccostrea kegaki*): Characterization of some genetic markers. *Zoolog. Sci.* **25**, 455–464 (2008).

25. O. V. Yurchenko, O. I. Skiteva, E. E. Voronezhskaya, V. A. Dyachuk, Nervous system development in the Pacific oyster, *Crassostrea gigas* (Mollusca: Bivalvia). *Front. Zool.* **15**, 10 (2018).

26. R. Feuda, I. S. Peter, Homologous gene regulatory networks control development of apical organs and brains in Bilateria. *Sci. Adv.* **8**, eabo2416 (2022).

27. A. Wanninger, Shaping the things to come: Ontogeny of lophotrochozoan neuromuscular systems and the tetraneuralia concept. *Biol. Bull.* **216**, 293–306 (2009).

28. L. Gąsiorowski, C. Andrikou, R. Janssen, P. Bump, G. E. Budd, C. J. Lowe, A. Hejnol, Molecular evidence for a single origin of ultrafiltration-based excretory organs. *Curr. Biol.* **31**, 3629–3638.e2 (2021).

29. Y. Kurita, N. Hashimoto, H. Wada, Evolution of the molluscan body plan: The case of the anterior adductor muscle of bivalves. *Biol. J. Linn. Soc.* **119**, 420–429 (2016).

30. S. L. Hooper, J. B. Thuma, Invertebrate muscles: Muscle specific genes and proteins. *Physiol. Rev.* **85**, 1001–1060 (2005).

31. V. Dyachuk, A. Wanninger, E. E. Voronezhskaya, Innervation of bivalve larval catch muscles by serotonergic and FMRFamidergic neurons. *Acta Biol. Hung.* **63** (Suppl. 2), 221–229 (2012).

32. V. Dyachuk, N. Odintsova, Development of the larval muscle system in the mussel Mytilus trossulus (Mollusca, Bivalvia). *Dev. Growth Differ.* **51**, 69–79 (2009).

33. J. E. Valencia, R. Feuda, D. O. Mellott, R. D. Burke, I. S. Peter, Ciliary photoreceptors in sea urchin larvae indicate pan-deuterostome cell type conservation. *BMC Biol.* **19**, 257 (2021).

34. P. Huan, G. Liu, H. Wang, B. Liu, Identification of a tyrosinase gene potentially involved in early larval shell biogenesis of the Pacific oyster *Crassostrea gigas*. *Dev. Genes Evol.* **223**, 389–394 (2013).

35. D. Zeng, X. Guo, Mantle transcriptome provides insights into biomineralization and growth regulation in the Eastern oyster (Crassostrea virginica). *Mar. Biotechnol.* **24**, 82–96 (2022).

36. X. Song, X. Wang, L. Li, G. Zhang, Identification two novel nacrein-like proteins involved in the shell formation of the Pacific oyster *Crassostrea gigas*. *Mol. Biol. Rep.* **41**, 4273–4278 (2014).

37. R. Zhao, T. Takeuchi, Y.-J. Luo, A. Ishikawa, T. Kobayashi, R. Koyanagi, A. Villar-Briones, L. Yamada, H. Sawada, S. Iwanaga, K. Nagai, N. Satoh, K. Endo, Dual gene repertoires for larval and adult shells reveal molecules essential for molluscan shell formation. *Mol. Biol. Evol.* **35**, 2751–2761 (2018).

38. D. J. Jackson, C. McDougall, K. Green, F. Simpson, G. Wörheide, B. M. Degnan, A rapidly evolving secretome builds and patterns a sea shell. *BMC Biol.* **4**, 40 (2006).

39. P. Rompolas, J. Azimzadeh, W. F. Marshall, S. M. King, Analysis of ciliary assembly and function in planaria. *Methods Enzymol.* **525**, 245–264 (2013).

40. M. L. Scimone, M. Srivastava, G. W. Bell, P. W. Reddien, A regulatory program for excretory system regeneration in planarians. *Development* **138**, 4387–4398 (2011).

41. C. Nielsen, Larval and adult brains. *Evol. Dev.* **7**, 483–489 (2005).

42. T. C. Lacalli, The nervous system and ciliary band of Müller's larva. *Proc. R. Soc. Lond. B Biol. Sci.* **217**, 37–58 (1982).

43. M. Marinković, J. Berger, G. Jékely, Neuronal coordination of motile cilia in locomotion and feeding. *Philos. Trans. R. Soc. Lond. B Biol. Sci.* **375**, 20190165 (2020).

44. C. Verasztó, N. Ueda, L. A. Bezares-Calderón, A. Panzera, E. A. Williams, R. Shahidi, G. Jékely, Ciliomotor circuitry underlying whole-body coordination of ciliary activity in the Platynereis larva. *eLife* **6**, e2600 (2017).

45. M. L. Scimone, O. Wurtzel, K. Malecek, C. T. Fincher, I. M. Oderberg, K. M. Kravarik, P. W. Reddien, FoxF-1 controls specification of non-body wall muscle and phagocytic cells in planarians. *Curr. Biol.* **28**, 3787–3801.e6 (2018).

46. A. J. Nederbragt, P. te Welscher, S. van den Driesche, A. E. van Loon, W. J. A. G. Dictus, Novel and conserved roles for *orthodenticle*/*otx* and *orthopedia*/*otp* orthologs in the gastropod mollusc *Patella vulgata*. *Dev. Genes Evol.* **212**, 330–337 (2002).

47. Y. Harada, N. Okai, S. Taguchi, K. Tagawa, T. Humphreys, N. Satoh, Developmental expression of the hemichordate otx ortholog. *Mech. Dev.* **91**, 337–339 (2000).

48. E. Shoguchi, Y. Harada, T. Numakunai, N. Satoh, Expression of the otx gene in the ciliary bands during sea cucumber embryogenesis. *Genesis* **27**, 58–63 (2000).

49. H. García-Castro, N. J. Kenny, M. Iglesias, P. Álvarez-Campos, V. Mason, A. Elek, A. Schönauer, V. A. Sleight, J. Neiro, A. Aboobaker, J. Permanyer, M. Irimia, A. Sebé-Pedrós, J. Solana, ACME dissociation: A versatile cell fixation-dissociation method for single-cell transcriptomics. *Genome Biol.* **22**, 89 (2021).

50. F. M. Martín-Zamora, Y. Liang, K. Guynes, A. M. Carrillo-Baltodano, B. E. Davies, R. D. Donnellan, Y. Tan, G. Moggioli, O. Seudre, M. Tran, K. Mortimer, N. M. Luscombe, A. Hejnol, F. Marlétaz, J. M. Martín-Durán, Annelid functional genomics reveal the origins of bilaterian life cycles. *Nature* **615**, 105–110 (2023).

51. K. Achim, N. Eling, H. M. Vergara, P. Y. Bertucci, J. Musser, P. Vopalensky, T. Brunet, P. Collier, V. Benes, J. C. Marioni, D. Arendt, Whole-body single-cell sequencing reveals transcriptional domains in the annelid larval body. *Mol. Biol. Evol.* **35**, 1047–1062 (2018).

52. R. Annunziata, M. Perillo, C. Andrikou, A. G. Cole, P. Martinez, M. I. Arnone, Pattern and process during sea urchin gut morphogenesis: The regulatory landscape. *Genesis* **52**, 251–268 (2014).

53. F. Xu, F. Marlétaz, D. Gavriouchkina, X. Liu, T. Sauka-Spengler, G. Zhang, P. W. H. Holland, Evidence from oyster suggests an ancient role for Pdx in regulating insulin gene expression in animals. *Nat. Commun.* **12**, 3117 (2021).

54. P. Paganos, P. Ronchi, J. Carl, G. Mizzon, P. Martinez, G. Benvenuto, M. I. Arnone, Integrating single cell transcriptomics and volume electron microscopy confirms the presence of pancreatic acinar-like cells in sea urchins. *Front. Cell Dev. Biol.* **10**, 991664 (2022).

55. P. R. H. Steinmetz, A. Aman, J. E. M. Kraus, U. Technau, Gut-like ectodermal tissue in a sea anemone challenges germ layer homology. *Nat. Ecol. Evol.* **1**, 1535–1542 (2017).

56. K. A. Rawlinson, The diversity, development and evolution of polyclad flatworm larvae. *Evodevo* **5**, 9 (2014).

57. P. R. H. Steinmetz, R. P. Kostyuchenko, A. Fischer, D. Arendt, The segmental pattern of otx, gbx, and Hox genes in the annelid *Platynereis dumerilii*. *Evol. Dev.* **13**, 72–79 (2011).

58. M. Conzelmann, E. A. Williams, K. Krug, M. Franz-Wachtel, B. Macek, G. Jékely, The neuropeptide complement of the marine annelid *Platynereis dumerilii*. *BMC Genomics* **14**, 906 (2013).

59. J. J. Henry, M. Q. Martindale, Conservation and innovation in spiralian development, in *Reproductive Strategies and Developmental Patterns in Annelids* (Springer Netherlands, 1999), pp. 255–265; http://dx.doi.org/10.1007/978-94-017-2887-4_15.

60. B. Hatschek, Studien über die Entwicklungsgsgeschichte der Anneliden. *Ein Beitrag zur Morphologie der Bilaterien*.

61. D. J. Leite, L. Piovani, M. J. Telford, Genome assembly of the polyclad flatworm *Prostheceraeus crozieri*. *Genome Biol. Evol.* **14**, evac133 (2022).

62. T. Stuart, A. Butler, P. Hoffman, C. Hafemeister, E. Papalexi, W. M. Mauck 3rd, Y. Hao, M. Stoeckius, P. Smibert, R. Satija, Comprehensive integration of single-cell data. *Cell* **177**, 1888–1902.e21 (2019).

63. J. M. Musser, K. J. Schippers, M. Nickel, G. Mizzon, A. B. Kohn, C. Pape, P. Ronchi, N. Papadopoulos, A. J. Tarashansky, J. U. Hammel, F. Wolf, C. Liang, A. Hernández-Plaza, C. P. Cantalapiedra, K. Achim, N. L. Schieber, L. Pan, F. Ruperti, W. R. Francis, S. Vargas, S. Kling, M. Renkert, M. Polikarpov, G. Bourenkov, R. Feuda, I. Gaspar, P. Burkhardt, B. Wang, P. Bork, M. Beck, T. R. Schneider, A. Kreshuk, G. Wörheide, J. Huerta-Cepas, Y. Schwab, L. L. Moroz, D. Arendt, Profiling cellular diversity in sponges informs animal cell type and nervous system evolution. *Science* **374**, 717–723 (2021).

64. C. C. Osborne, K. J. Perry, M. Shankland, J. Q. Henry, Ectomesoderm and epithelial-mesenchymal transition-related genes in spiralian development. *Dev. Dyn.* **247**, 1097–1120 (2018).

65. J. Barrera-Redondo, J. S. Lotharukpong, H.-G. Drost, S. M. Coelho, Uncovering gene-family founder events during major evolutionary transitions in animals, plants and fungi using GenEra. *Genome Biol.* **24**, 54 (2023).

66. T. Domazet-Loso, J. Brajković, D. Tautz, A phylostratigraphy approach to uncover the genomic history of major adaptations in metazoan lineages. *Trends Genet.* **23**, 533–539 (2007).

67. A. L. De Oliveira, A. Calcino, A. Wanninger, Extensive conservation of the proneuropeptide and peptide prohormone complement in mollusks. *Sci. Rep.* **9**, 4846 (2019).

68. D. Thiel, M. Franz-Wachtel, F. Aguilera, A. Hejnol, Xenacoelomorph neuropeptidomes reveal a major expansion of neuropeptide systems during early bilaterian evolution. *Mol. Biol. Evol.* **35**, 2528–2543 (2018).

69. D. Thiel, L. A. Yañez-Guerra, M. Franz-Wachtel, A. Hejnol, G. Jékely, Nemertean, brachiopod, and phoronid neuropeptidomics reveals ancestral spiralian signaling systems. *Mol. Biol. Evol.* **38**, 4847–4866 (2021).

70. V. A. Dyachuk, Hematopoiesis in Bivalvia larvae: Cellular origin, differentiation of hemocytes, and neoplasia. *Dev. Comp. Immunol.* **65**, 253–257 (2016).

71. J. Li, Y. Zhang, Y. Liu, Y. Zhang, Z. Xiang, F. Qu, Z. Yu, A thymosin beta-4 is involved in production of hemocytes and immune defense of Hong Kong oyster, *Crassostrea hongkongensis*. *Dev. Comp. Immunol.* **57**, 1–9 (2016).

72. N. R. de la Ballina, A. Villalba, A. Cao, Differences in proteomic profile between two haemocyte types, granulocytes and hyalinocytes, of the flat oyster *Ostrea edulis*. *Fish Shellfish Immunol.* **100**, 456–466 (2020).

73. C. Barreau-Roumiguière, C. Montagnani, J. M. Escoubas, Characterization of a Tal/SCL-like transcription factor in the pacific oyster *Crassostrea gigas*. *Dev. Comp. Immunol.* **27**, 793–800 (2003).

74. D. H. Parma, P. E. Bennett Jr., R. E. Boswell, Mago Nashi and Tsunagi/Y14, respectively, regulate *Drosophila* germline stem cell differentiation and oocyte specification. *Dev. Biol.* **308**, 507–519 (2007).

75. M. Thiruvalluvan, P. G. Barghouth, A. Tsur, L. Broday, N. J. Oviedo, SUMOylation controls stem cell proliferation and regional cell death through Hedgehog signaling in planarians. *Cell. Mol. Life Sci.* **75**, 1285–1301 (2018).

76. H. Orii, T. Sakurai, K. Watanabe, Distribution of the stem cells (neoblasts) in the planarian *Dugesia japonica*. *Dev. Genes Evol.* **215**, 143–157 (2005).

77. G. T. Eisenhoffer, H. Kang, A. Sánchez Alvarado, Molecular analysis of stem cells and their descendants during cell turnover and regeneration in the planarian *Schmidtea mediterranea*. *Cell Stem Cell* **3**, 327–339 (2008).
